# Associations of urinary phytoestrogen biomarkers with uric acid and hyperuricemia, and the mediating role of kidney function

**DOI:** 10.1186/s12937-025-01241-2

**Published:** 2025-11-12

**Authors:** Min Luan, Youping Tian, Xianfeng Wu, Kuangyang Chen, Cheng Hu

**Affiliations:** 1 Shanghai Diabetes Institute, Shanghai Key Laboratory of Diabetes Mellitus, Shanghai Clinical Centre for Diabetes, Shanghai Sixth People’s Hospital, Shanghai Jiao Tong University School of Medicine, Shanghai, China; 2 National Management Office of Neonatal Screening Project for Congenital Heart Disease (CHD), Children’s Hospital of Fudan University, National Children’s Medical Center, Shanghai, China; 3 Department of Nephrology, Shanghai Jiao Tong University Affiliated Sixth People’s Hospital, Shanghai, China; 4 Department of Endocrinology, The Second Affiliated Hospital, School of Medicine, Zhejiang University, Hangzhou, Zhejiang China

**Keywords:** Uric acid, Hyperuricemia, Phytoestrogen biomarker, Kidney function, NHANES

## Abstract

**Background:**

Hyperuricemia is increasingly acknowledged as a major public health issue. Current evidence on the effects of urinary phytoestrogen biomarkers on hyperuricemia is limited. Moreover, the potential mediation effect of kidney function was not assessed.

**Methods:**

This study included 2,793 adults aged 20–79 years from the National Health and Nutrition Examination Surveys (NHANES 2007–2010). We used traditional regression and Bayesian kernel machine regression (BKMR) models to assess the associations of urinary phytoestrogen biomarkers with serum uric acid and hyperuricemia risk, and the mediation effect model to evaluate the role of kidney function in their associations. Estimated glomerular filtration rate (eGFR) was calculated for assessing kidney function.

**Results:**

Most phytoestrogen biomarkers were inversely associated with serum uric acid and hyperuricemia risk, with mean changes in the estimates ranging from − 0.06 to -0.12 mg/dl and odds ratios ranging from 0.80 to 0.90. The BKMR model demonstrated that higher urinary concentrations of phytoestrogen mixtures were associated with lower serum uric acid and hyperuricemia risk, and identified that equol (EQU) and enterolactone (ENT) were the two most important contributors to the overall inverse associations. The BKMR model further confirmed that EQU and ENT were independently associated with lower serum uric acid and hyperuricemia risk. Causal mediation analysis indicated eGFR mediated the associations of EQU and ENT with hyperuricemia risk, with the proportion of mediation ranging from 9.64 to 11.60% (all *P* < 0.05), respectively.

**Conclusions:**

Urinary phytoestrogen biomarkers were inversely associated with serum uric acid levels and the risk of hyperuricemia, with EQU and ENT identified as the major contributing metabolites. These findings provide preliminary evidence that renal function may partially mediate the associations of EQU and ENT with uric acid concentrations and hyperuricemia risk.

**Supplementary Information:**

The online version contains supplementary material available at 10.1186/s12937-025-01241-2.

## Introduction

Uric acid is the end product of purine metabolism in the humans. Both the overproduction of serum uric acid and impaired renal urate excretion are main causes of hyperuricemia [1], which is widely recognized as a risk factor for chronic diseases, such as gout, hypertension, type 2 diabetes, chronic kidney disease, and cardiovascular disease [2–4]. The global prevalence of hyperuricemia ranges from 2.6% to 36% across different populations, posing a significant public health concern [5]. Moreover, effective curative options remain limited [6]. Therefore, identifying potential dietary factors that may help balance serum uric acid is critical to reducing the risk of hyperuricemia.

Phytoestrogens are a group of plant-derived bioactive compounds, and are classified into four classes: isoflavones, lignans, coumestans, and stilbenes [7]. Isoflavones and lignans are the two main classes of phytoestrogens found in foods, such as soybeans, oilseeds, and legumes [8, 9]. Isoflavones and lignans are primarily ingested as glycosides and metabolized by gut bacteria in the large intestine into their bioactive phytoestrogen metabolites, with isoflavones converted into genistein (GEN), daidzein (DAD), O-desmethylangolensin (O-DMA), and equol (EQU) [10], and lignans transformed into enterolactone (ENT) and enterodiol (END) [11]. Given their structural similarity to endogenous estrogens, phytoestrogens may influence uric acid metabolism through estrogen-related pathways, including the inhibition of xanthine oxidase and the regulation of renal tubular urate transporters, thereby affecting both uric acid production and excretion [12, 13]. Numerous human studies have evaluated the association between soy consumption and uric acid levels, but findings have been inconsistent [9, 14]. Most previous studies used a food frequency questionnaire to collect habitual diet, which could have been subject to measurement error and dietary recall bias. Although several studies have examined the effects of the active ingredients of purified soy isoflavones on uric acid levels, inter-individual differences in isoflavone metabolism may further contribute to these inconsistencies [15, 16]. Urinary phytoestrogens show reasonable performance in measuring habitual intake of isoflavones and lignans [17], serving as a reliable biomarker of dietary phytoestrogen intake. However, the association between urinary phytoestrogen biomarkers and uric acid levels and hyperuricemia risk remain unclear.

Uric acid is primarily eliminated by the kidney, and impaired kidney function can disrupt the uric acid balance, resulting in hyperuricemia [18]. Evidence from animal studies has indicated that polyphenolic compounds can regulate renal organic anion transporters, which may contribute to decrease the risk of hyperuricemia in mice [14]. Organic anion transporters are essential proteins that mediate urate transport from the blood to intracellular tubular cells [15]. However, no population-based studies have focused on whether phytoestrogen exposure affects uric acid levels by modulating kidney function. To address this gap, we applied mediation analysis, which allows for investigating the mediating role of kidney function in the association between urinary phytoestrogen exposure and hyperuricemia risk. By integrating biomarker-based exposure measurements with a formal mediation framework, our study provides insights to understand how phytoestrogens may affect uric acid metabolism. Therefore, the present study aimed to assess the associations of urinary phytoestrogen biomarkers with uric acid and the risk of hyperuricemia in the US adults, and evaluated the potential role of kidney function in their associations.

## Materials and methods

### Study design and population

**Fig. 1:**
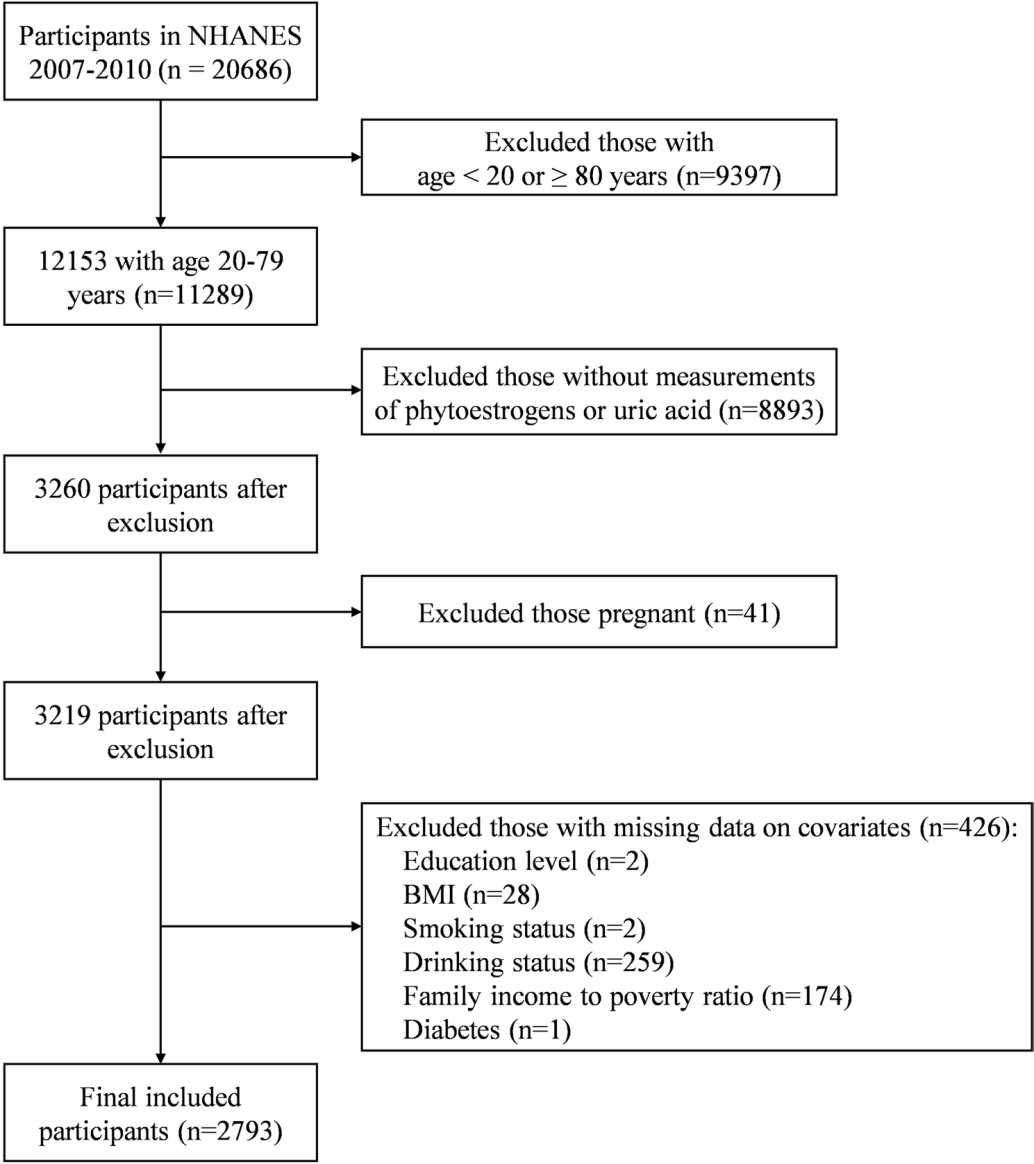
Study population of the present study from the NHANES 2007-2010

We used publicly available data from the NHANES 2007–2010. A total of 11,289 adults aged between 20 and 79 years were enrolled, 3,260 of whom had available data on serum uric acid and urinary phytoestrogen concentrations. Adults who were pregnant (*n* = 41) and those with missing covariate data (*n* = 426) were excluded. Finally, 2,793 adults with complete data on serum uric acid, urinary phytoestrogens, and other covariates were included, as shown in Fig. 1. All procedures of the NHANES were reviewed and approved by the Research Ethics Review Committee of the NCHS.

### Phytoestrogen measurements

Six phytoestrogens, including four isoflavones (DAD, O-DMA, EQU and GEN) and two lignans (ENT and END), were measured in urine using utilizing high-performance liquid chromatography–atmospheric pressure photoionization–tandem mass spectrometry [16]. The limits of detection (LODs) were 0.4 ng/mL for DAD, 0.2 ng/mL for O-DMA and GEN, 0.06 ng/mL for EQU, 0.1 ng/mL for ENT, and 0.04 ng/mL for END. Urinary phytoestrogen concentrations below the LOD were replaced with the LOD divided by the square root of 2 [19]. To correct for urine dilution, phytoestrogen concentrations were standardized to the creatinine concentration and are presented as µg/g creatinine [20]. Urinary creatinine was measured via the Roche ModP.

### Serum uric acid and hyperuricemia

Serum uric acid levels were measured via the Beckman UniCel^®^ DxC800 Synchron via the timed endpoint method. We defined hyperuricemia as serum uric acid levels of ≥ 7 mg/dl for males and ≥ 6 mg/dl for females [21].

### Kidney function assessment

We used the Chronic Kidney Disease Epidemiology Collaboration (CKD-EPI) creatinine-cystatin C formula to calculate the estimated glomerular filtration rate (eGFR, mL·min^−1^·1.73 m^−2^) for assessing kidney function [22].

#### Covariates

Covariates were selected from previous studies on the relationship between nutrient intake and serum uric acid. We also considered the variables associated with serum uric acid or hyperuricemia risk in bivariate analyses (Table 1 and Supplementary Table S1, *P* value < 0.10). Participants’ age (continuous), sex, race/ethnicity (Mexican American, other Hispanic, Non-Hispanic White, Non-Hispanic Black, and other races), educational level (less than high school, high school graduate/GED or equivalent, and college or above), BMI (under/normal weight: < 25 kg/m^2^, overweight: 25–30 kg/m^2^, and obesity: ≥ 30 kg/m^2^), poverty income ratio (PIR) (≤ 1.30, 1.30–1.85 and ≥ 1.85), recreational physical activity (vigorous, moderate and inactive), smoking status (yes and no), drinking status (yes and no), diabetes status (yes and no), and hypertension status (yes and no) were included in the final models. Diabetes was defined as meeting one or more of the following criteria: glycohemoglobin A1c (HbA1c) >6.5%, fasting blood glucose >126 mg/dL, 2-hours plasma glucose ≥ 200 mg/dl, and self-report of a diagnosis by the patient’s healthcare provider [23]. Hypertension was described as having a diagnosis by a doctor or other health professional with high blood pressure, taking prescribed medicine for high blood pressure or having systolic blood pressure level of ≥ 130 mm Hg or diastolic blood pressure level of ≥ 80 mm Hg. Individuals aged ≥ 20 years with albumin-creatinine ratio ≥ 30 mg/g or eGFR < 60 ml/min/1.73 m^2^ were considered to have CKD [24].

**Table 1:** Weighted characteristics of the study population

Characteristics	Overall (*N* = 2793)	Hyperuricemia		*P*-value
		No (*N* = 2217)	Yes (*N* = 576)	
Age (mean ± SD, years)	45.07 ± 0.54	45.07 ± 0.59	48.81 ± 0.63	< 0.001
Sex (n, %)				
Male	1394 (49.25)	1071 (47.07)	323 (58.07)	< 0.001
Female	1399 (50.75)	1146 (52.93)	253 (41.93)	
Race (n, %)				
Mexican American	558 (9.19)	477 (9.8)	81 (6.72)	0.045
Other Hispanic	276 (4.56)	228 (4.8)	48 (3.61)	
Non-Hispanic White	1313 (69.72)	1034 (69.37)	279 (71.14)	
Non-Hispanic Black	528 (10.76)	382 (10.09)	146 (13.47)	
Other Races	118 (5.77)	96 (5.95)	22 (5.06)	
Body mass index (BMI) categories (n, %)				
Normal	765 (31.08)	699 (35.58)	66 (12.79)	< 0.001
Overweight	935 (32.24)	779 (34.12)	156 (24.59)	
Obesity	1093 (36.69)	739 (30.3)	354 (62.63)	
Educational level (n, %)				
Less than high school	302 (5.35)	247 (5.45)	55 (4.94)	0.652
High school graduate/GED or equivalent	469 (12.66)	366 (12.44)	103 (13.59)	
College or above	2022 (81.99)	1604 (82.12)	418 (81.47)	
Family monthly poverty level category (n, %)				
≤1.30	970 (23.16)	781 (23.31)	189 (22.54)	0.430
1.30–1.85	402 (12.30)	317 (11.84)	85 (14.16)	
≥1.85	1421 (64.54)	1119 (64.85)	302 (63.29)	
Recreational physical activities (n, %)				
No	1481 (46.05)	1139 (44.75)	342 (51.32)	0.011
Moderate	577 (25.7)	481 (26.91)	96 (20.77)	
Vigorous	735 (28.25)	597 (28.34)	138 (27.92)	
Smoking status (n, %)				
No	1442 (51.83)	1166 (51.96)	276 (51.3)	0.840
Yes	1351 (48.17)	1051 (48.04)	300 (48.7)	
Drinking status (n, %)				
No	711 (21.44)	574 (21.86)	137 (19.71)	0.338
Yes	2082 (78.56)	1643 (78.14)	439 (80.29)	
Diabetes (n, %)				
No	2467 (92.01)	1996 (93.53)	471 (85.83)	< 0.001
Yes	326 (7.99)	221 (6.47)	105 (14.17)	
Hypertension (n, %)				
No	1359 (53.57)	1189 (57.94)	170 (35.82)	< 0.001
Yes	1434 (46.43)	1028 (42.06)	406 (64.18)	
CKD (n, %)				
No	2406 (90.16)	1969 (91.67)	437 (84.07)	< 0.001
Yes	387 (9.84)	248 (8.33)	139 (15.93)	
Survey year (n, %)				
2007–2008	1310 (48.78)	1034 (48.73)	276 (49)	0.934
2009–2010	1483 (51.22)	1183 (51.27)	300 (51)	

### Statistical analyses

The statistical analyses for this study incorporated sample weights, clustering, and stratification due to the complex multi-stage stratified probability survey design employed in NHANES. Specifically, a specific sampling weight for phytoestrogen (laboratory subsample B) was constructed for the complete dataset by employing data from two combined 2-year cycles. We described the demographic characteristics of the participants with and without hyperuricemia by mean ± standard deviation (SD) for continuous variables and counts (percentages) for categorical variables. Urinary creatinine-corrected phytoestrogen concentrations were natural log (ln) transformed to approximate a normal distribution. We used *Pearson* correlation to examine the correlations between pairs of ln-transformed urinary phytoestrogen biomarkers. Given that a few nonlinear associations were indicated in the restricted cubic spline (*P* value < 0.10, Supplementary Figure S1 and Figure S2), urinary phytoestrogen concentrations were categorized into four groups according to quartiles in further analyses. Sampling-weighted multivariable linear models were used for evaluating the relationships between urinary phytoestrogen concentrations and uric acid levels. Sampling-weighted binary logistic regression analyses were performed to calculate the odds ratios (OR) and 95% confidence intervals (CIs) for exposures on hyperuricemia risk. Three models were performed: Model 1 was the crude model; Model 2 adjusted for sociodemographic covariates including age, sex, race/ethnicity, poverty income ratio, body mass index, and educational level; Model 3 was further adjusted for smoking status, alcohol consumption, recreational physical activity, and history of hypertension and diabetes.

We further applied Bayesian kernel machine regression (BKMR) models to evaluate the overall effects of exposure to the six phytoestrogens on serum uric acid and hyperuricemia risk, along with their respective contributions [25, 26]. BKMR performs regression of the exposure-response function using a Gaussian kernel function iteratively, which allows exploration of nonlinear dose-response relationships between phytoestrogen mixtures and hyperuricemia, as well as interactions among phytoestrogens. The model could assess the associations between exposure and continuous variables or binary outcome (1/0) variables. In this study, the BKMR model was fitted using the Markov Chain Monte Carlo (MCMC) with 10,000 iterations. According to the chemical structure of phytoestrogens, we grouped GEN, DAD, O-DMA and EQU into group 1, and ENT and END into group 2. To identify the most important phytoestrogens within the mixture, we presented the group posterior inclusion probability (groupPIP) and conditional posterior inclusion probability (condPIP) from BKMR. The group PIP indicated the most important class of phytoestrogens within the mixture, and cond. PIP indicated the most important phytoestrogen within a class of phytoestrogens. PIP values range from 0 to 1, and 0.5 is usually considered the threshold value [27, 28]. We presented the overall effect of the phytoestrogen mixture by calculating the expected change in uric acid or hyperuricemia risk for departures of all phytoestrogens in the mixture from their 25th percentile level. The single-exposure effect was estimated as the difference in uric acid levels or hyperuricemia risk associated with an interquartile range (IQR) increase in the concentration of a given phytoestrogen, while the other phytoestrogens are fixed at the 25th, 50th, or 75th percentile levels.

A causal mediation analysis was performed using the “mediation” R package to evaluate whether kidney function mediates the association between phytoestrogen exposure and hyperuricemia risk. In this model, the most influential phytoestrogen identified by the BKMR analysis was treated as the exposure, hyperuricemia as the outcome, and eGFR as the mediator. The mediation framework assumes that the exposure–outcome, exposure–mediator, and mediator–outcome associations are all statistically significant. The direct effect represents the influence of phytoestrogen exposure on hyperuricemia risk independent of kidney function, while the indirect effect reflects the pathway through kidney function. The proportion mediated was calculated to quantify the extent to which the anti-hyperuricemic effect of phytoestrogen exposure is explained by kidney function [29].We conducted a series of sensitivity analyses. First, we repeated analyses using an alternative definition of hyperuricemia based on the American College of Rheumatology guideline threshold (serum urate ≥ 7.0 mg/dL) [30]. Second, to ensure that our main findings are free from potential measured confounders, we performed stratified analyses by sex and survey cycle. We also repeated the main analyses within subgroups with a potentially low risk of hyperuricemia, including adults with normal weight; those who were not smokers and drinkers; or those who had no history of hypertension, diabetes, or CKD. We further adjusted for the total protein intake to control for the influence of dietary factors. We also identified the most important contributors to serum uric acid levels (continuous outcomes) in the BKMR models. To address potential creatinine coupling, in which both eGFR and urinary phytoestrogens involve creatinine, we repeated the main and mediation analyses considering the exposure as unadjusted urinary concentrations and including urinary creatinine as a covariate.

BKMR model, mediation analyses, and sensitivity analyses were conducted with adjustment for the covariates included in Model 3. Statistical analyses were conducted using SAS 9.4 (SAS Institute Inc., Cary, NC, USA) and R 4.2.2 (R Development Core Team). The mediation effect model was conducted using the “mediation” package. A *P*-value < 0.05 from two-tailed tests was considered statistically significant.

## Results

### Study participants

Among the 2,793 adults included, 576 (20.62%) were diagnosed with hyperuricemia. The mean (SD) age was 45.07 (0.54) years. Most participants were overweight or obese (68.93%), had a college education or higher (81.99%), and reported alcohol consumption (78.56%). Compared with adults without hyperuricemia, those with hyperuricemia were more likely to be older, obese, have diabetes, hypertension, or CKD, and were less likely to engage in physical activity (Table 1).

### Distributions of urinary phytoestrogen concentrations

Six phytoestrogens were detected in almost all the urine samples of U.S. adults (> 90%). ENT had the highest median concentration, followed by DAD and END (Table 2). EQU, GEN, O-DMA, and DAD were moderately to highly correlated (r ranging from 0.24 to 0.87, *P* value < 0.0001; Supplementary Figure S3). END concentration was moderately correlated with ENT (*r* = 0.53, *P* < 0.0001). No correlation was found between GEN and ENT.

**Table 2:** Distributions of urinary phytoestrogen biomarkers

	Detection rates (%)	GM (GSD)	5th	25th	50th	75th	95th
Phytoestrogen							
DAD	99.89	60.66 (6.23)	3.90	16.70	48.60	196.00	1570.00
O-DMA	93.02	4.03 (10.69)	0.14	0.60	2.60	19.10	300.00
EQU	99.68	6.18 (4.45)	0.64	2.63	5.98	13.30	53.90
GEN	100.00	29.33 (5.71)	2.40	8.30	24.10	85.10	634.00
ENT	99.82	223.13 (6.34)	6.40	79.00	298.00	844.00	2550.00
END	99.32	34.52 (5.97)	1.59	14.10	40.40	103.00	450.00
Creatinine-corrected phytoestrogen							
DAD	-	62.76 (5.70)	5.53	17.41	48.26	188.66	1456.69
O-DMA	-	4.17 (10.34)	0.18	0.68	2.78	19.20	364.84
EQU	-	6.40 (4.09)	0.81	2.94	6.02	12.67	48.03
GEN	-	30.34 (5.27)	3.24	8.84	22.97	83.33	655.56
ENT	-	230.85 (6.28)	6.55	87.87	329.95	838.30	2442.31
END	-	35.71 (5.67)	2.06	15.08	41.27	101.82	402.06

*Abbreviations*: *DAD* Daidzein, *O-DMA* O-Desmethylangolensin, *EQU* Equol, *GEN* Genistein, *ETD* Enterodiol, *ENT* Enterolactone, *GM* Geometric mean, *GSD* Geometric standard deviation

### Associations of phytoestrogens with serum uric acid and hyperuricemia risk

The inverse associations of most urinary phytoestrogens with serum uric acid levels and hyperuricemia risk were consistently observed across Model 1 to Model 3 (Table 3&Supplementary Table S2). Specially, each unit increase in ln-transformed concentrations of DAD, O-DMA, EQU, and ENT was associated with a decrease of 0.06 mg/dl (95% CI: −0.09, −0.04), 0.07 mg/dl (95% CI: −0.08, −0.05), 0.12 mg/dl (95% CI: −0.16, −0.07), and 0.08 mg/dl (95% CI: −0.11, −0.05) respectively in serum uric acid levels. When hyperuricemia was examined as a binary outcome, each unit increase in ln-transformed concentrations of DAD, O-DMA, EQU, and ENT was associated with lower risk of hyperuricemia (DAD: relative risk (OR) = 0.90, 95% CI: 0.84, 0.96; O-DMA: OR = 0.85, 95% CI: 0.80, 0.90; EQU: OR = 0.80, 95% CI: 0.70, 0.92; ENT: OR = 0.85, 95% CI: 0.79, 0.91, Table 3). When individuals were further stratified into four subgroups based on quartile levels of phytoestrogens, similar patterns of inverse associations between phytoestrogen exposure and serum uric acid levels, as well as hyperuricemia risk, were observed. Compared with those in the lowest quartile, individuals in the highest quartile concentrations of O-DMA, EQU, and ENT had both lower uric acid levels and hyperuricemia risk (Table 3). Besides, we also found that the highest quartile concentration of END was inversely associated with uric acid levels and hyperuricemia.

**Table 3:** Associations of urinary phytoestrogen biomarkers with serum uric acid concentrations and hyperuricemia risk in sampling-weighted regression models

	Serum uric acid		Hyperuricemia	
	β (95% CI)	*P*-value	OR (95% CI)	*P*-value
DAD				
Quartile1	Ref	Ref	Ref	
Quartile2	−0.14 (−0.31, 0.03)	0.105	1.02 (0.65, 1.59)	0.948
Quartile3	−0.26 (−0.39, −0.13)	< 0.001	0.8 (0.57, 1.13)	0.197
Quartile4	−0.30 (−0.45, −0.16)	< 0.001	0.7 (0.47, 1.04)	0.075
Continuous	−0.06 (−0.09, −0.04)	< 0.001	0.90 (0.84, 0.96)	0.002
O-DMA				
Quartile1	Ref	Ref	Ref	Ref
Quartile2	−0.33 (−0.53, −0.14)	0.002	0.56 (0.38, 0.84)	0.007
Quartile3	−0.42 (−0.58, −0.26)	< 0.001	0.53 (0.37, 0.75)	0.001
Quartile4	−0.45 (−0.58, −0.31)	< 0.001	0.42 (0.28, 0.63)	< 0.001
Continuous	−0.07 (−0.08, −0.05)	< 0.001	0.85 (0.80, 0.90)	< 0.001
EQU				
Quartile1	Ref	Ref	Ref	Ref
Quartile2	−0.16 (−0.28, −0.04)	0.011	0.83 (0.61, 1.12)	0.221
Quartile3	−0.23 (−0.4, −0.06)	0.011	0.74 (0.45, 1.22)	0.230
Quartile4	−0.49 (−0.68, −0.31)	< 0.001	0.46 (0.31, 0.69)	< 0.001
Continuous	−0.12 (−0.16, −0.07)	< 0.001	0.80 (0.70, 0.92)	< 0.001
GEN				
Quartile1	Ref	Ref	Ref	Ref
Quartile2	−0.23 (−0.38, −0.07)	0.005	0.83 (0.58, 1.19)	0.305
Quartile3	−0.08 (−0.24, 0.09)	0.365	1.13 (0.76, 1.66)	0.539
Quartile4	−0.2 (−0.32, −0.08)	0.001	0.84 (0.61, 1.15)	0.263
Continuous	−0.02 (−0.05, −0.00)	0.046	0.99 (0.93, 1.05)	0.689
END				
Quartile1	Ref	Ref	Ref	Ref
Quartile2	−0.07 (−0.23, 0.08)	0.333	0.72 (0.5, 1.04)	0.078
Quartile3	−0.1 (−0.23, 0.03)	0.115	0.74 (0.54, 1.02)	0.063
Quartile4	−0.14 (−0.26, −0.02)	0.026	0.65 (0.43, 0.99)	0.046
Continuous	−0.02 (−0.04, 0.01)	0.185	0.95 (0.88, 1.03)	0.218
ENT				
Quartile1	Ref	Ref	Ref	Ref
Quartile2	−0.30 (−0.46, −0.13)	0.001	0.53 (0.36, 0.79)	0.003
Quartile3	−0.32 (−0.47, −0.18)	< 0.001	0.49 (0.36, 0.67)	< 0.001
Quartile4	−0.33 (−0.46, −0.19)	< 0.001	0.49 (0.34, 0.72)	< 0.001
Continuous	−0.08 (−0.11, −0.05)	< 0.001	0.85 (0.79, 0.91)	0.001

*Abbreviations*: *DAD* Daidzein, *O-DMA* O-desmethylangolensin, *EQU* Equol, *GEN* Genistein, *END* Enterodiol, *ENT* Enterolactone

We adjusted for age, sex, race/ethnicity, poverty income ratio, body mass index, educational level, smoking status, alcohol consumption, recreational physical activity, hypertension, and diabetes

### BKMR models

**Fig. 2:**
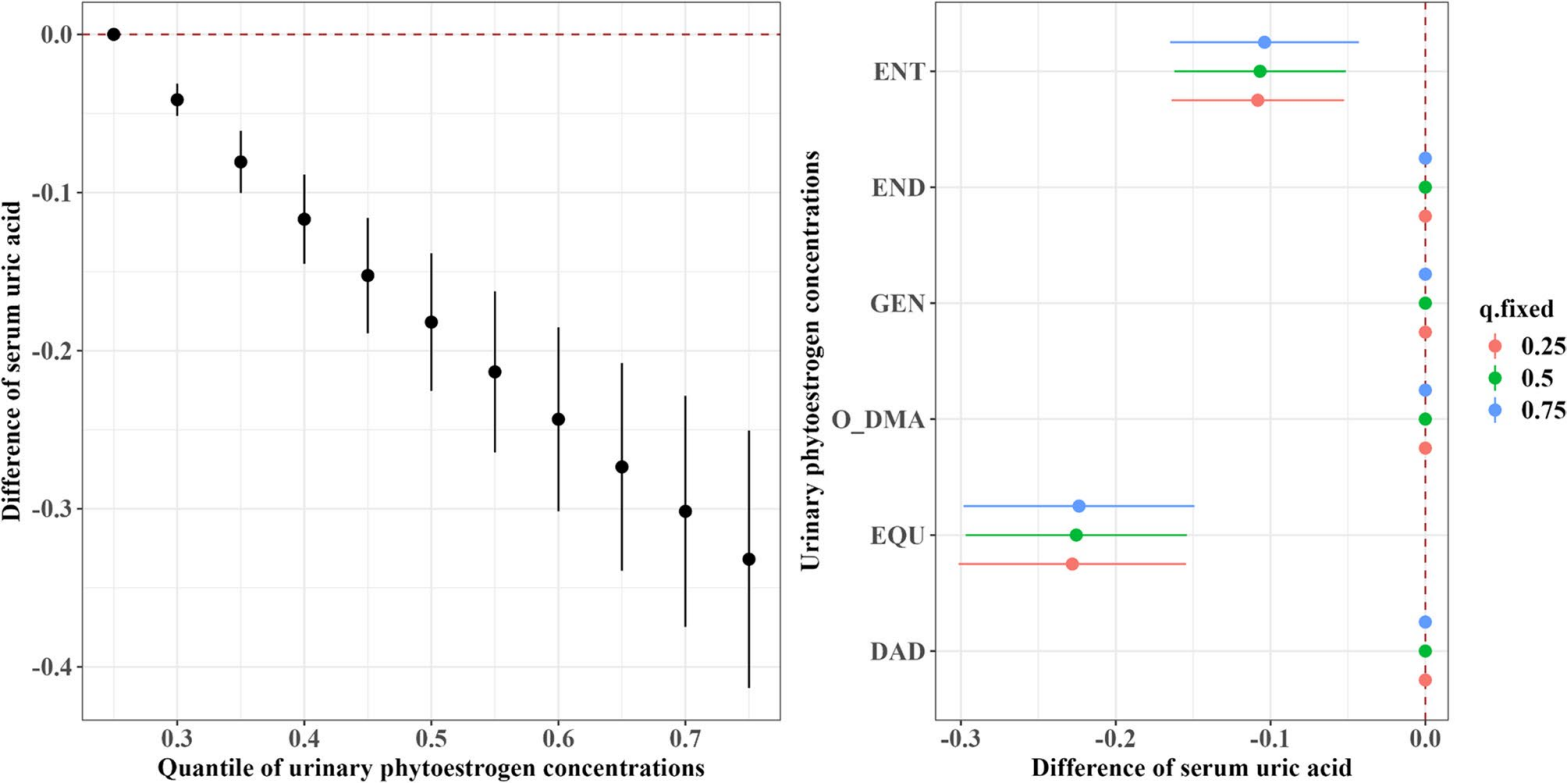
Overall and single-exposure effects of urinary phytoestrogen biomarkers on serum uric acid levels (continuous) in the Bayesian kernel machine regression models. Higher phytoestrogen mixture concentrations were associated with lower serum uric acid; the urinary EQU and ENT concentrations were independently associated with the lower serum uric acid levels. Left: overall associations of urinary phytoestrogen concentrations with serum uric acid; right: single associations of urinary phytoestrogen concentrations with serum uric acid. We adjusted for age, sex, race/ethnicity, poverty income ratio, body mass index, educational level, smoking status, alcohol consumption, recreational physical activity, hypertension, and diabetes. Abbreviations: DAD, daidzein; O-DMA, O-desmethylangolensin; EQU, equol; GEN, genistein; END, enterodiol; ENT, enterolactone. Note: The overall effect of the phytoestrogen mixture was presented by calculating the expected change in uric acid for departures of all phytoestrogens in the mixture from their 25th percentile level. The single-exposure effect refers to the association of an interquartile range increase in a particular phytoestrogen with uric acid when the remaining phytoestrogens are concurrently fixed at specific percentile levels

In the BKMR model, higher urinary phytoestrogen mixtures were associated with lower serum uric acid levels (Fig. 2). Compared with the 25th percentile, the 75th percentile concentration of the phytoestrogen mixtures was associated with a − 0.33 mg/dL (95% credible intervals (CrI): − 0.41, − 0.25) decrease in serum uric acid. Among the phytoestrogen groups, isoflavones (groupPIP = 1.00) contributed most strongly to the observed effect, with EQU identified as the predominant driver within the isoflavones (condPIP = 1.00; Supplementary Table S3). An IQR increase in EQU was associated with ~–0.23 mg/dL lower serum uric acid. Similar inverse associations between ENT and serum uric acid were also observed (Fig. 2).

**Fig. 3:**
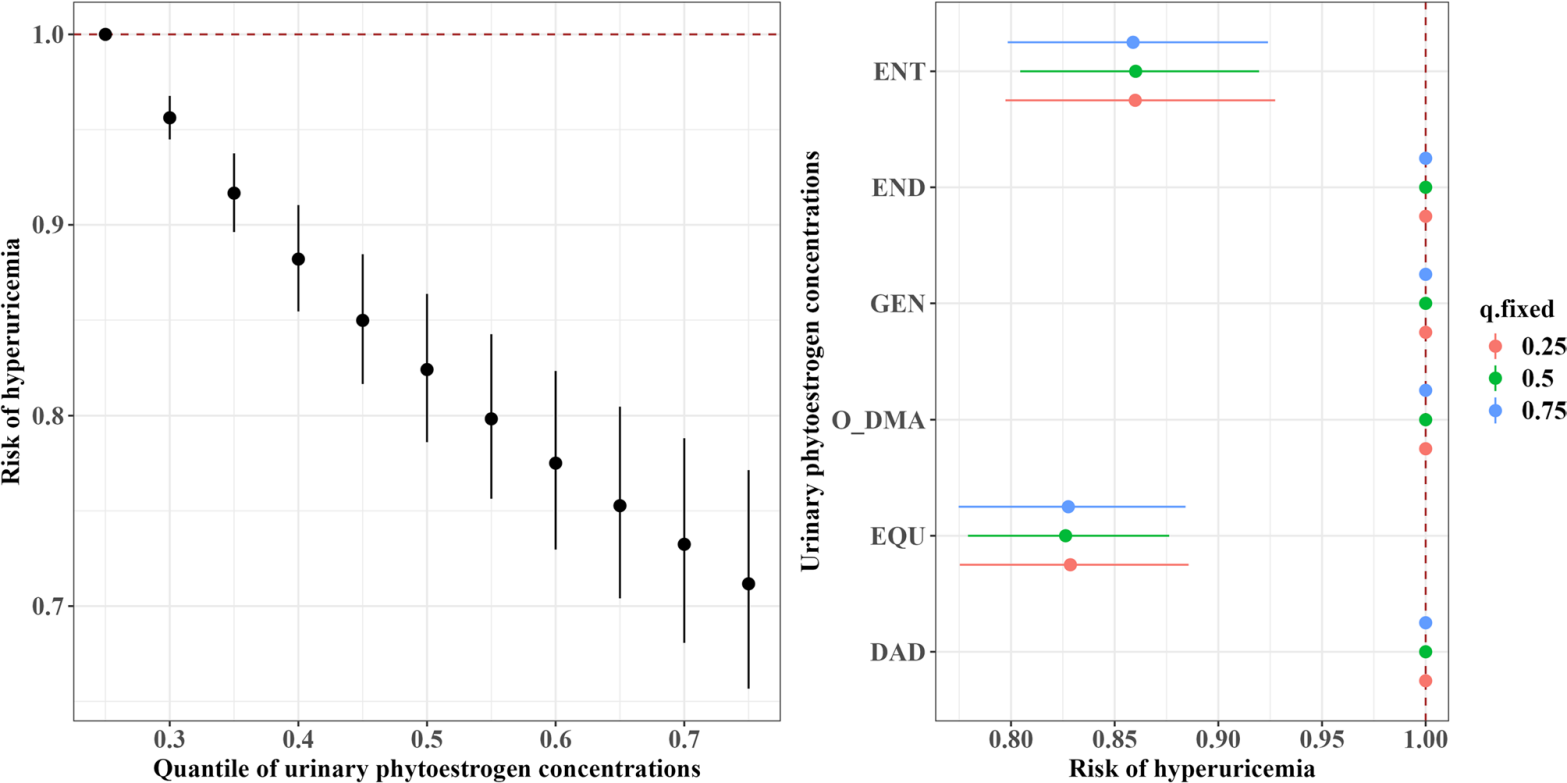
Overall and single associations of urinary phytoestrogen biomarkers with the risk of hyperuricemia in the Bayesian kernel machine regression model. Higher phytoestrogen mixture concentrations were associated with lower risk of hyperuricemia; the urinary EQU and ENT concentrations were independently associated with the lower risk of hyperuricemia. Left: overall associations of urinary phytoestrogen concentrations with the risk of hyperuricemia; right: single associations of urinary phytoestrogen concentrations with the risk of hyperuricemia. We adjusted for age, sex, race/ethnicity, poverty income ratio, body mass index, educational level, smoking status, alcohol consumption, recreational physical activity, hypertension, and diabetes. Abbreviations: DAD, daidzein; O-DMA, O-desmethylangolensin; EQU, equol; GEN, genistein; END, enterodiol; ENT, enterolactone. Note: The overall effect of the phytoestrogen mixture was presented by calculating the expected change in hyperuricemia risk for departures of all phytoestrogens in the mixture from their 25th percentile level. The single-exposure effect refers to the association of an interquartile range increase in a particular phytoestrogen with hyperuricemia risk when the remaining phytoestrogens are concurrently fixed at specific percentile levels

For hyperuricemia, the BKMR model also showed an overall inverse association of phytoestrogen mixtures with hyperuricemia risk (Fig. 3). Compared with the 25th percentile, the 75th percentile concentration of phytoestrogen mixtures was associated with a 29% lower risk of hyperuricemia (OR = 0.71, 95% CrI: 0.66, 0.77). EQU and ENT remained independently associated with reduced risk when other phytoestrogens were fixed at different percentiles. Consistently, EQU and ENT were identified as the main contributors within the isoflavones and lignans groups, respectively (condPIP = 1.00 and 1.00, Supplementary Table S3).

### Mediation analyses

**Fig. 4:**
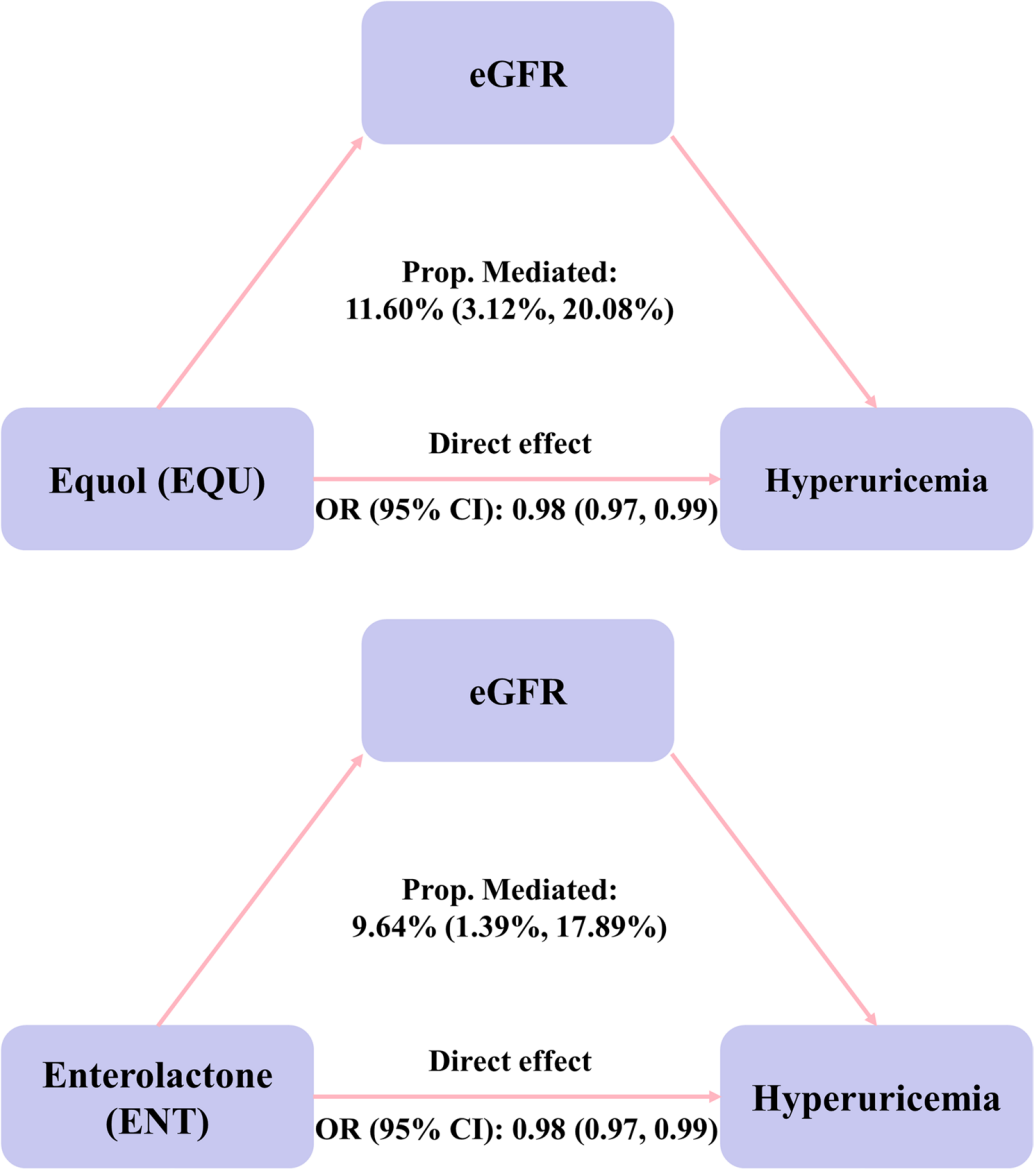
Mediation role of eGFR in the associations of equol and enterolactone with hyperuricemia risk. eGFR played a mediating role in the associations of EQU and ENT with hyperuricemia risk, with mediation effects of 11.6% and 9.64%, respectively. The model adjusted for age, sex, race/ethnicity, the poverty income ratio, body mass index, educational level, smoking status, alcohol consumption, recreational physical activity, hypertension, and diabetes

Because the independent effects of EQU and ENT were indicated, we considered whether eGFR mediated the association between EQU or ENT and hyperuricemia risk. Each unit increase in the ln-transformed concentrations of EQU and ENT was associated with an increase of 0.57 (95% CI: 0.18, 0.97) and 0.36 (95% CI: 0.06, 0.67) units in eGFR, respectively (Supplementary Table S4). Notably, eGFR had significant mediated effects on the associations of EQU and ENT with hyperuricemia risk, and the proportion of mediation was 11.60% (95% CI: 3.12%, 20.08%) and 9.64% (95% CI: 1.39%, 17.89%), respectively (both *P* < 0.05; Fig. 4).

### Sensitivity analyses

When we used the American College of Rheumatology guideline threshold for hyperuricemia (serum urate ≥ 7.0 mg/dL), stratified analyses by sex or survey cycle, restricted subgroup analyses without overweight, smoking, alcohol consumption, histories of hypertension, diabetes, and CKD in the models, as well as additionally adjusted for total protein intake the inverse association between urinary phytoestrogen biomarkers and uric acid levels and hyperuricemia risk remained. (Supplementary Table S5-S8). After repeating the analyses considering the exposure as unadjusted urinary concentrations and including urinary creatinine as a covariate, we observed similar results (Supplementary Table S9 & Figure S4).

## Discussion

In the present study, urinary phytoestrogen biomarkers were inversely associated with serum uric acid and hyperuricemia risk. We also found an inverse association between phytoestrogen mixture exposure and hyperuricemia risk, with EQU and ENT being the most important contributors. Our findings provide preliminary evidence that kidney function mediated the associations of urinary phytoestrogen with hyperuricemia risk. These findings were strengthened when similar associations were observed using various analytical strategies.

Previous population-based studies have reported inconclusive associations between dietary soy foods, soy products, or isoflavones and serum uric acid levels or hyperuricemia risk, including positive, null, and inverse findings [8, 31, 32]. These inconsistencies may stem from misclassification bias in recall-based dietary questionnaires. In contrast to studies relying on dietary recall, our investigation, which utilized urinary phytoestrogen biomarkers, reported an inverse association between phytoestrogen exposure and both serum uric acid levels and hyperuricemia risk. In line with our results, a randomized controlled trial from China [33] reported that purified DAD supplementation was associated with decreased uric acid, though another trial reported no effect [34]. One possible explanation for variant responsiveness to DAD may be differences in the metabolism of DAD among individuals, specifically variation in EQU and/or O-DMA-synthesising capacity [35, 36]. Notably, our study identified EQU as the most significant contributor to the observed decrease in uric acid levels among isoflavone metabolites. This is consistent with a previous small study, which found reduced uric acid levels exclusively in the EQU-producing group [36]. Furthermore, epidemiological evidence indicates that higher urinary EQU levels, but not those of other isoflavones, are associated with improved cardiometabolic health, providing further support for a distinctive role of EQU [37, 38]. Lignans, another class of phytoestrogens abundant in oilseeds, whole grains, and other fiber-rich plant foods, also exhibit antioxidant and anti-inflammatory properties [39]. Only one study by Zhuo et al. [40] reported that dietary lignans were inversely associated with hyperuricemia risk. We additionally reported that ENT, other than END, was the most important contributor to decreased risk of hyperuricemia among lignan metabolites. These findings underscore the importance of addressing population heterogeneity in EQU and END production, as inter-individual differences may modulate the metabolic and health benefits of soy intake. The differences in the metabolism of phytoestrogens are influenced by sociodemographic factors (e.g., ethnicity, age, education), lifestyle factors (e.g., dietary habits), and biological factors (e.g., gut microbiota composition or enzymatic activity) [41–43]. Specifically, EQU-producing prevalence is estimated at 20–30% in Western populations but tends to be higher in Asian populations [41, 42]. Future studies should take interindividual metabolic variability into account when examining the health effects of isoflavone intake.

We further associated the findings with hyperuricemia and observed that eGFR mediated approximately 9.64–11.60% of the association between urinary phytoestrogen exposure and hyperuricemia. These findings suggest a potential mechanistic pathway whereby phytoestrogen exposure may be inversely associated with hyperuricemia through kidney function. The kidneys are the primary organs responsible for uric acid excretion [31, 44]. Phytoestrogens, due to their structural similarity to endogenous estrogens, can bind to estrogen receptors (ERα and ERβ) and exert estrogenic effects. The effects of estrogen on the kidneys in both animal models and human studies are well documented [12]. For example, estrogen deficiency in animal models has been shown to induce structural and functional renal impairment [12]. Consistently, we observed that phytoestrogen exposure was associated with higher eGFR, which may preserve renal function and thereby promote uric acid clearance, reducing hyperuricemia risk. In addition, phytoestrogens may directly influence renal urate handling. Experimental studies have shown that polyphenolic compounds can modulate organic anion transporters, key mediators of tubular urate transport, thereby reducing hyperuricemia risk in mice [13].

This study has several strengths. First, the present study is the first to evaluate the associations of urinary phytoestrogen biomarkers with serum uric acid concentrations and hyperuricemia. This method of internal exposure assessment considers individual differences in phytoestrogen metabolism. Second, the present study initially compared the relative importance of various phytoestrogens, including prototypes and their metabolites. Our findings provide new insights, revealing that EQU, a bioactive isoflavone metabolite, was the most significant contributor associated with the effects of soy food consumption on hyperuricemia, followed by ENT, a lignan metabolite.

Several limitations must be noted when our findings are interpreted. First, the cross-sectional design of the epidemiological analysis does not allow for causal conclusions. Second, our analyses were limited by the reliance on a single spot urine sample to characterize exposure to phytoestrogen. However, phytoestrogen concentrations in a single urine sample have been shown to exhibit solid temporal reliability over several months, suggesting that a single measurement can serve as a reliable biomarker of dietary phytoestrogen intake [13, 45]. Nevertheless, the potential misclassification caused by the single-spot measurement should be non-differential and may have null-attenuated the effect estimates. Third, given the cross-sectional nature of our study, exposure, mediator, and outcome were measured at the same time, which did not fulfill the requirements of temporal sequencing for mediation analysis. Moreover, the mediation effect was relatively modest. Therefore, our findings provided only preliminary evidence for the mediating role of kidney function in the association between exposure and hyperuricemia. Given the limitations in the current study, these results need to be interpreted with caution and further investigations are needed to support our findings. Fourth, although the associations remained robust when we adjusted for a wide range of dietary and lifestyle factors, we cannot fully rule out the possibility of residual confounding from specific dietary components, particularly plant-based protein sources.

In conclusion, urinary phytoestrogen concentrations were associated with decreased uric acid levels and hyperuricemia risk, and EQU and ENT were identified as the most important contributors. Our findings provided preliminary evidence that kidney function mediated the associations of urinary phytoestrogen with hyperuricemia risk.

## Supplementary Information

Supplementary Material 1

## Data Availability

This study was carried out using publicly available data from the NHANES official website: [https://wwwn.cdc.gov/nchs/nhanes/Default.aspx]. The code and other data used for this study are available from the corresponding author upon reasonable request.

## Abbreviations

DAD: daidzein
O-DMA: O-desmethylangolensin
EQU: equol
GEN: genistein
END: enterodiol
ENT: enterolactone
