# Supplementary material for: Associations of urinary phytoestrogen biomarkers with uric acid and hyperuricemia, and the mediating role of kidney function
Source: Nutr J. 2025 Nov 12;24:171. doi: 10.1186/s12937-025-01241-2 (PMC12613696; doi:10.1186/s12937-025-01241-2)
Supplement: Supplementary file 1 — Supplementary Material 1 [file 12937_2025_1241_MOESM1_ESM.docx]

**Associations of urinary phytoestrogen biomarkers with uric acid and hyperuricemia, and the mediating role of kidney function**

Min Luan^1^, Youping Tian^2^, Xianfeng Wu^3^, Kuangyang Chen^4^, Cheng Hu^1,*^

^1^Shanghai Diabetes Institute, Shanghai Key Laboratory of Diabetes Mellitus, Shanghai Clinical Centre for Diabetes, Shanghai Sixth People's Hospital Affiliated to Shanghai Jiao Tong University School of Medicine, Shanghai, China.

^2^National Management Office of Neonatal Screening Project for Congenital Heart Disease (CHD), Children's Hospital of Fudan University, National Children's Medical Center, Shanghai, China.

^3^Department of Nephrology, Shanghai Jiao Tong University Affiliated Sixth People's Hospital, Shanghai, China

^4^Department of Endocrinology, The Second Affiliated Hospital, School of Medicine, Zhejiang University, Hangzhou, Zhejiang, China

**^*^ Correspondence to:**

Cheng Hu, Shanghai Diabetes Institute, Shanghai Key Laboratory of Diabetes Mellitus, Shanghai Clinical Centre for Diabetes, Shanghai Sixth People's Hospital Affiliated with Shanghai Jiao Tong University School of Medicine, Shanghai 200233, China.

E-mail: [alfredhc@sjtu.edu.cn](mailto:alfredhc@sjtu.edu.cn)

**Contents of supplementary materials**

**Figure S1.** Associations of ln-transformed concentrations of urinary phytoestrogen biomarkers with serum uric acid in restricted cubic spline models

**Figure S2.** Associations of ln-transformed concentrations of urinary phytoestrogen biomarkers with hyperuricemia in restricted cubic spline models

**Figure S3.** Pearson correlation coefficients between pairs of urinary phytoestrogen biomarkers

**Figure S4.** eGFR played a mediating role in the associations of EQU and ENT with hyperuricemia risk

**Table S1.** Univariate analyses of participants’ characteristics with serum uric acid concentrations

**Table S2.** Associations of urinary phytoestrogen biomarkers with serum uric acid concentrations and hyperuricemia risk (model 1 and 2).

**Table S3.** Posterior inclusion probabilities for group inclusion into models and conditional inclusion into models using Bayesian kernel machine regression model.

**Table S4.** Associations of urinary phytoestrogen biomarkers with the estimated glomerular filtration rate.

**Table S5.** Associations of urinary phytoestrogen biomarkers with hyperuricemia according to the American College of Rheumatology guidelines in multiple logistic regression models

**Table S6.** Associations of urinary phytoestrogen biomarkers with serum uric acid concentrations and hyperuricemia risk stratified by sex and survey cycle

**Table S7.** Associations of urinary phytoestrogen biomarkers with serum uric acid concentrations and hyperuricemia risk within different subgroups

**Table S8.** Associations of urinary phytoestrogen biomarkers with serum uric acid concentrations and hyperuricemia risk, additionally adjusted for total protein intake

**Table S9.** Associations of uncorrected urinary phytoestrogen biomarkers with serum uric acid concentrations and hyperuricemia risk, with urinary creatinine as an adjustment covariate


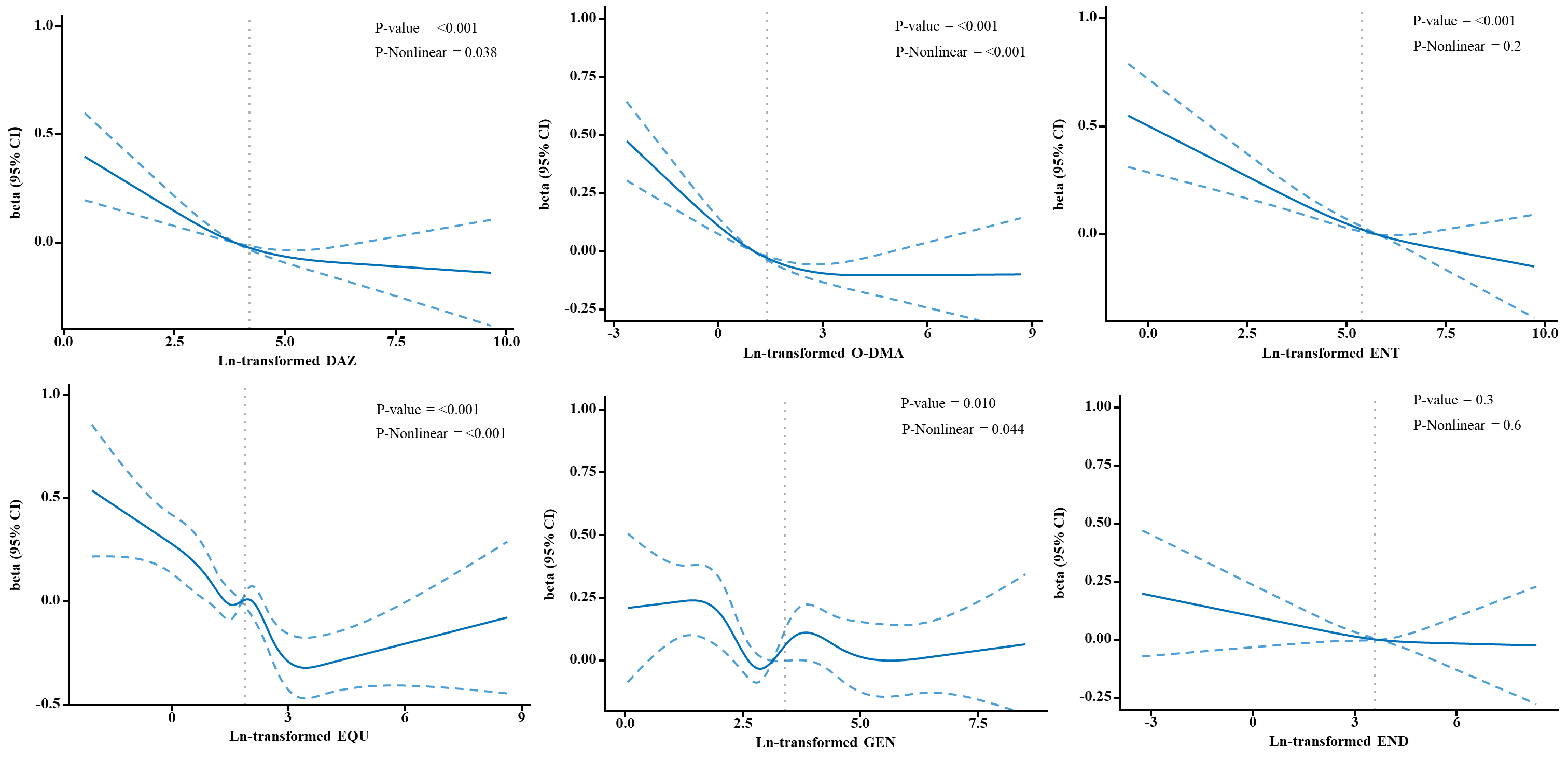


**Figure S1** Associations of ln-transformed concentrations of urinary phytoestrogen biomarkers with serum uric acid in restricted cubic spline models

Adjusting for Adjusting for age, sex, race/ethnicity, poverty income ratio, body mass index, educational levels, smoking status, alcohol consumption, recreational physical activity, hypertension, and diabetes.


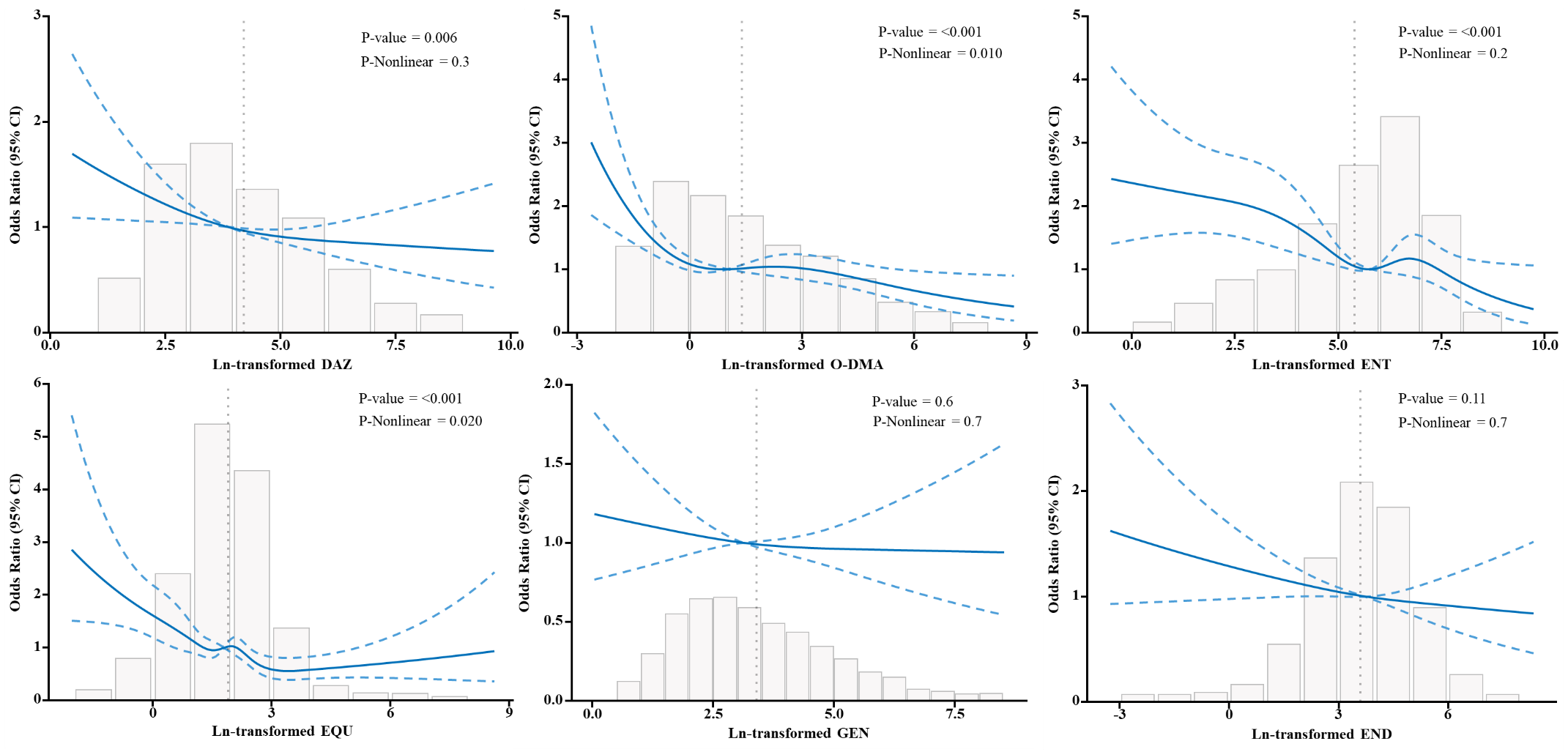


**Figure S2** Associations of ln-transformed concentrations of urinary phytoestrogen biomarkers with hyperuricemia in restricted cubic spline models

Adjusting for Adjusting for age, sex, race/ethnicity, poverty income ratio, body mass index, educational levels, smoking status, alcohol consumption, recreational physical activity, hypertension, and diabetes.


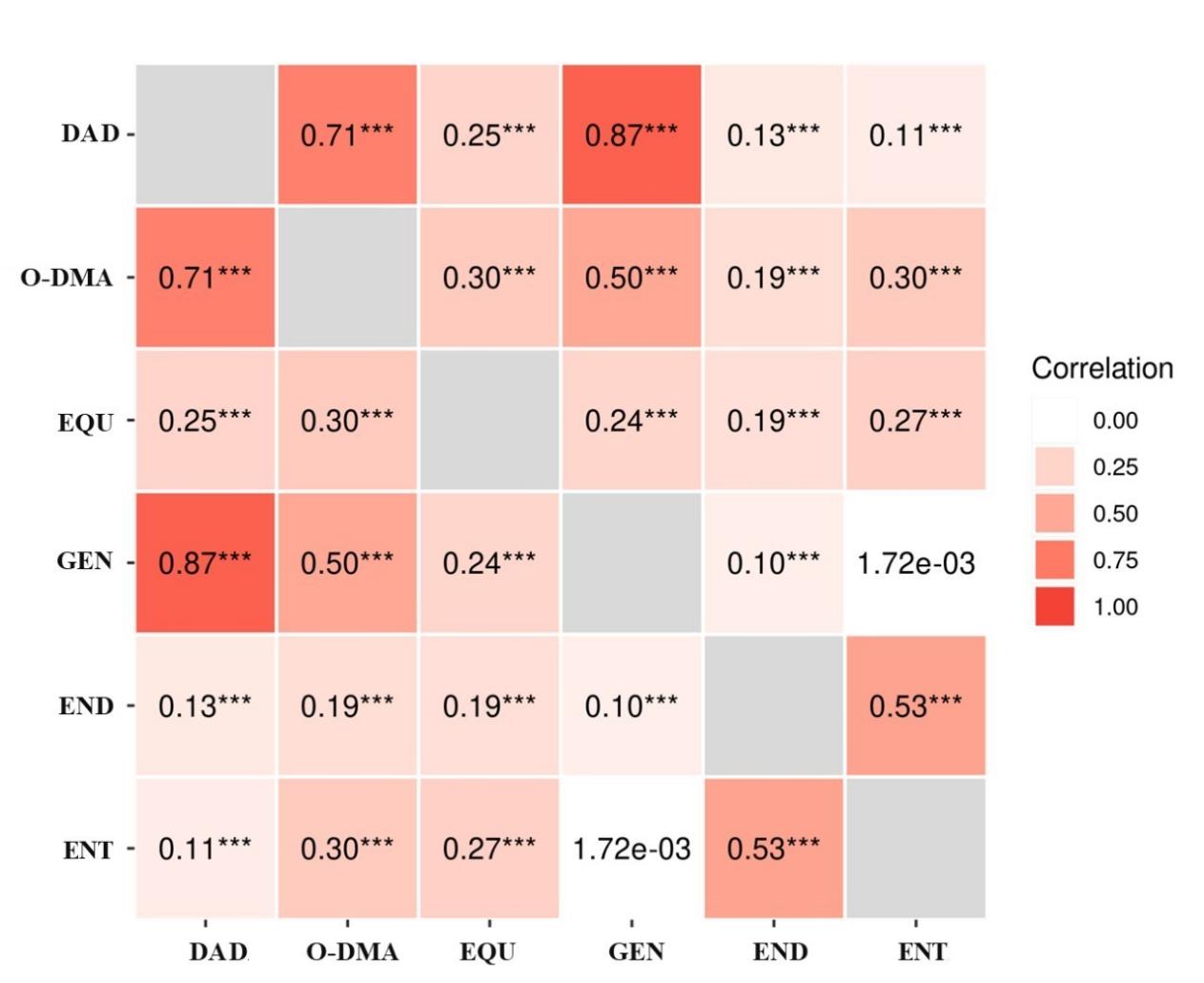


**Figure S3** Pearson correlation coefficients between pairs of urinary phytoestrogen biomarkers

Note: Abbreviations: DAD, daidzein; O-DMA, O-desmethylangolensin; EQU, equol; GEN, genistein; ETD, enterodiol; ENT, enterolactone

**
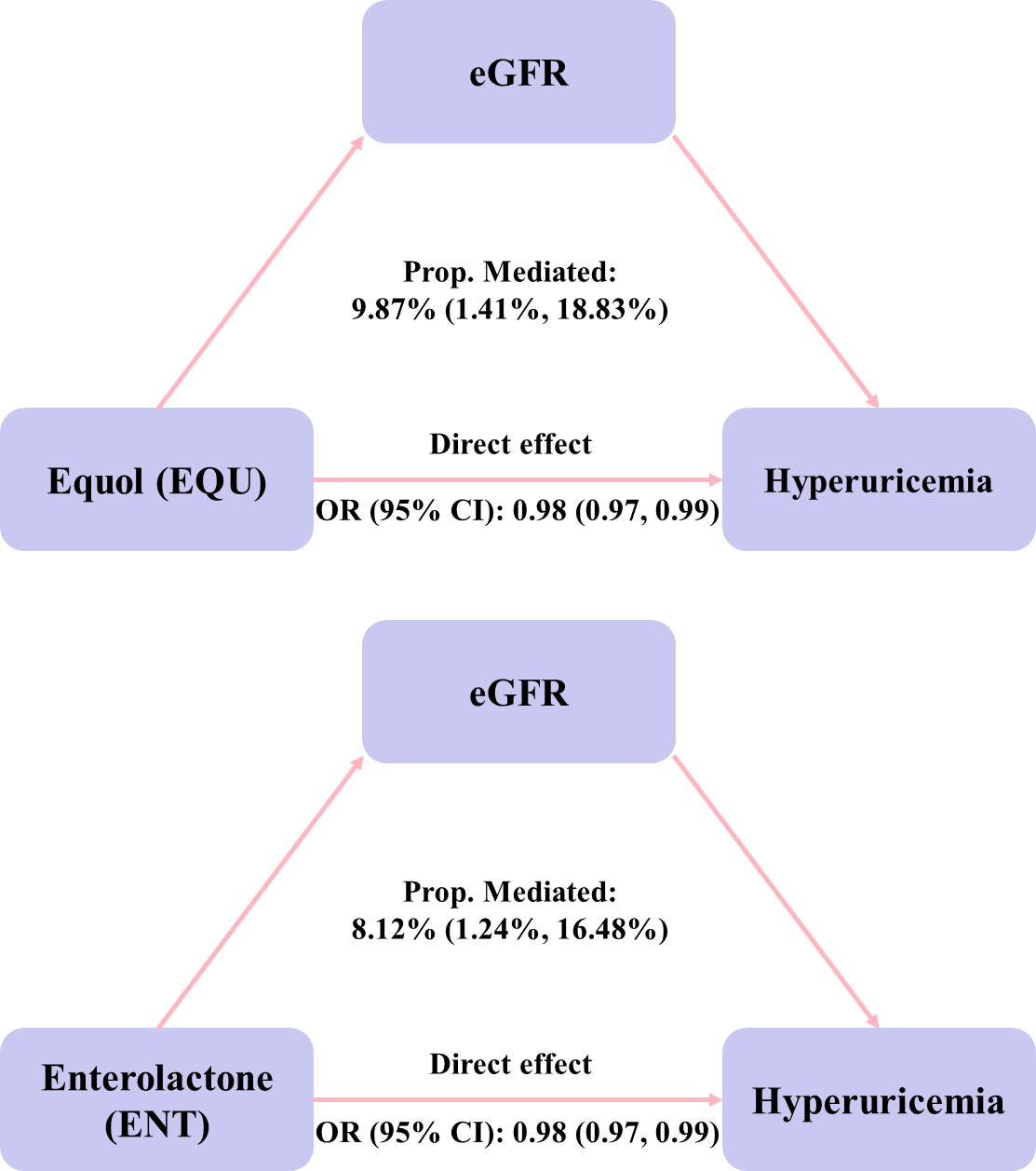
Figure S4.** Mediation role of eGFR in the associations of uncorrected equol and enterolactone with hyperuricemia risk.

Model was adjusted for age, sex, race/ethnicity, poverty income ratio, body mass index, educational level, smoking status, alcohol consumption, recreational physical activity, hypertension, urine creatinine, and diabetes.

**Table S1** Univariate analyses of participants’ characteristics with serum uric acid concentrations

| **Characteristics** | **Serum uric acid (mean ± SD)** | ***P*-value** |
| --- | --- | --- |
| Sex |  |  |
| Male | 6.11 ± 1.29 | <0.001 |
| Female | 4.89 ± 1.29 |  |
| Race |  |  |
| Mexican American | 5.25 ± 1.29 | <0.001 |
| Other Hispanic | 5.43 ± 1.51 |  |
| Non-Hispanic White | 5.51 ± 1.40 |  |
| Non-Hispanic Black | 5.77 ± 1.52 |  |
| Other Races | 5.47 ± 1.38 |  |
| Body mass index (BMI) categories | |  |
| Normal | 4.93 ± 1.29 | <0.001 |
| Overweight | 5.43 ± 1.33 |  |
| Obesity | 5.95 ± 1.44 | |
| Educational level |  |  |
| Less than high school | 5.45 ± 1.35 | 0.779 |
| High school graduate /GED or equivalent | 5.48 ± 1.39 |  |
| College or above | 5.51 ± 1.44 | |
| Family monthly poverty level category | |  |
| ≤1.30 | 5.42 ± 1.44 | 0.091 |
| 1.30–1.85 | 5.54 ± 1.44 |  |
| ≥1.85 | 5.54 ± 1.40 | |
| Recreational activities | |  |
| No | 5.52 ± 1.37 | 0.066 |
| Moderate | 5.40 ± 1.30 |  |
| Vigorous | 5.54 ± 1.49 |  |
| Smoking status |  |  |
| No | 5.38 ± 1.40 | <0.0001 |
| Yes | 5.63 ± 1.44 | |
| Drinking status |  |  |
| No | 5.25 ± 1.40 | <0.0001 |
| Yes | 5.59 ± 1.42 | |
| Diabetes |  |  |
| No | 5.46 ± 1.38 | <0.001 |
| Yes | 5.77 ± 1.65 |  |
| Hypertension |  |  |
| No | 5.14 ± 1.26 | <0.0001 |
| Yes | 5.84 ± 1.48 | |
| Survey year |  |  |
| 2007–2008 | 5.52 ± 1.38 | 0.505 |
| 2009–2010 | 5.48 ± 1.46 | |

Table S2. Associations of urinary phytoestrogen biomarkers with serum uric acid concentrations and hyperuricemia risk (model 1 and 2).

|  | **Serum uric acid** | | | |  | **Hyperuricemia** | | | |
| --- | --- | --- | --- | --- | --- | --- | --- | --- | --- |
|  | Crude model 1  β (95% CI) | *P*-value | Basic-adjusted model 2  β (95% CI) | *P*-value |  | Crude model  OR (95% CI) | *P*-value | Basic-adjusted  model 2  OR (95% CI) | *P*-value |
| Daidzein |  |  |  |  |  |  |  |  |  |
| Quartile1 | Ref | Ref | Ref | Ref |  | Ref | Ref | Ref | Ref |
| Quartile2 | -0.13 (-0.28, 0.02) | 0.084 | -0.10 (-0.23, 0.02) | 0.110 |  | 1.01 (0.79, 1.30) | 0.910 | 1.01 (0.77, 1.31) | 0.967 |
| Quartile3 | -0.17 (-0.32, -0.02) | 0.025 | -0.19 (-0.32, -0.06) | 0.004 |  | 0.82 (0.63, 1.06) | 0.131 | 0.75 (0.58, 0.99) | 0.041 |
| Quartile4 | -0.42 (-0.57, -0.28) | <0.001 | -0.26 (-0.39, -0.13) | <0.001 |  | 0.73 (0.56, 0.95) | 0.020 | 0.77 (0.58, 1.01) | 0.062 |
| Continuous | -0.09 (-0.12, -0.06) | <0.001 | -0.06 (-0.09, -0.04) | <0.001 |  | 0.91 (0.87, 0.96) | <0.001 | 0.91 (0.86, 0.97) | 0.002 |
| O-Desmethylangolensin | | | | | | | | |  |
| Quartile1 | Ref | Ref | Ref | Ref |  | Ref | Ref | Ref | Ref |
| Quartile2 | -0.31 (-0.46, -0.16) | <0.001 | -0.25 (-0.37, -0.12) | <0.001 |  | 0.71 (0.55, 0.91) | 0.007 | 0.68 (0.52, 0.88) | 0.004 |
| Quartile3 | -0.36 (-0.51, -0.21) | <0.001 | -0.35 (-0.47, -0.22) | <0.001 |  | 0.67 (0.52, 0.86) | 0.002 | 0.59 (0.45, 0.77) | <0.001 |
| Quartile4 | -0.50 (-0.65, -0.35) | <0.001 | -0.33 (-0.46, -0.20) | <0.001 |  | 0.59 (0.45, 0.76) | <0.001 | 0.58 (0.44, 0.77) | <0.001 |
| Continuous | -0.09 (-0.11, -0.06) | <0.001 | -0.06 (-0.08, -0.04) | <0.001 |  | 0.90 (0.87, 0.94) | <0.001 | 0.89 (0.85, 0.93) | <0.001 |
| Equol |  |  |  |  |  |  |  |  |  |
| Quartile1 | Ref | Ref | Ref | Ref |  | Ref | Ref | Ref | Ref |
| Quartile2 | -0.31 (-0.46, -0.16) | <0.001 | -0.27 (-0.39, -0.14) | <0.001 |  | 0.70 (0.55, 0.90) | 0.006 | 0.66 (0.51, 0.86) | 0.002 |
| Quartile3 | -0.37 (-0.51, -0.22) | <0.001 | -0.28 (-0.40, -0.15) | <0.001 |  | 0.70 (0.55, 0.90) | 0.005 | 0.69 (0.53, 0.89) | 0.005 |
| Quartile4 | -0.68 (-0.83, -0.53) | <0.001 | -0.52 (-0.65, -0.39) | <0.001 |  | 0.44 (0.34, 0.58) | <0.001 | 0.43 (0.32, 0.57) | <0.001 |
| Continuous | -0.16 (-0.20, -0.13) | <0.001 | -0.12 (-0.15, -0.08) | <0.001 |  | 0.81 (0.75, 0.87) | <0.001 | 0.79 (0.73, 0.86) | <0.001 |
| Genistein |  |  |  |  |  |  |  |  |  |
| Quartile1 | Ref | Ref | Ref | Ref |  | Ref | Ref | Ref | Ref |
| Quartile2 | -0.24 (-0.39, -0.10) | 0.001 | -0.19 (-0.31, -0.06) | 0.004 |  | 0.81 (0.62, 1.04) | 0.101 | 0.84 (0.64, 1.1) | 0.212 |
| Quartile3 | -0.12 (-0.27, 0.02) | 0.100 | -0.07 (-0.19, 0.06) | 0.306 |  | 0.97 (0.75, 1.24) | 0.798 | 1.01 (0.77, 1.31) | 0.952 |
| Quartile4 | -0.35 (-0.50, -0.20) | <0.001 | -0.19 (-0.32, -0.07) | 0.003 |  | 0.76 (0.59, 0.99) | 0.039 | 0.83 (0.63, 1.09) | 0.178 |
| Continuous | -0.07 (-0.10, -0.03) | <0.001 | -0.03 (-0.06, -0.01) | 0.018 |  | 0.95 (0.90, 1.01) | 0.100 | 0.97 (0.91, 1.03) | 0.335 |
| Enterodiol |  |  |  |  |  |  |  |  |  |
| Quartile1 | Ref | Ref | Ref | Ref |  | Ref | Ref | Ref | Ref |
| Quartile2 | -0.07 (-0.22, 0.08) | 0.356 | -0.10 (-0.22, 0.03) | 0.142 |  | 0.81 (0.63, 1.05) | 0.106 | 0.73 (0.56, 0.96) | 0.024 |
| Quartile3 | -0.10 (-0.25, 0.05) | 0.173 | -0.02 (-0.15, 0.11) | 0.729 |  | 0.91 (0.70, 1.16) | 0.435 | 0.91 (0.70, 1.18) | 0.480 |
| Quartile4 | -0.38 (-0.53, -0.23) | 0.000 | -0.07 (-0.20, 0.06) | 0.268 |  | 0.66 (0.50, 0.86) | 0.002 | 0.75 (0.57, 1.00) | 0.048 |
| Continuous | -0.07 (-0.10, -0.04) | <0.001 | -0.02 (-0.05, 0.00) | 0.081 |  | 0.93 (0.88, 0.98) | 0.006 | 0.94 (0.89, 0.99) | 0.022 |
| Enterolactone |  |  |  |  |  |  |  |  |  |
| Quartile1 | Ref | Ref | Ref | Ref |  | Ref | Ref | Ref | Ref |
| Quartile2 | -0.26 (-0.41, -0.11) | <0.001 | -0.21 (-0.33, -0.08) | 0.001 |  | 0.60 (0.47, 0.78) | <0.001 | 0.63 (0.49, 0.82) | 0.001 |
| Quartile3 | -0.34 (-0.49, -0.19) | <0.001 | -0.26 (-0.38, -0.13) | <0.001 |  | 0.59 (0.46, 0.75) | <0.001 | 0.60 (0.46, 0.78) | <0.001 |
| Quartile4 | -0.53 (-0.68, -0.38) | <0.001 | -0.21 (-0.34, -0.08) | 0.001 |  | 0.50 (0.38, 0.64) | <0.001 | 0.64 (0.49, 0.85) | 0.002 |
| Continuous | -0.11 (-0.14, -0.08) | <0.001 | -0.08 (-0.10, -0.05) | <0.001 |  | 0.86 (0.82, 0.91) | <0.001 | 0.86 (0.81, 0.9) | <0.001 |

Model 2 adjusted for age, sex, race/ethnicity, poverty income ratio, body mass index, and educational level.

**Table S3** Posterior inclusion probabilities for group inclusion into models and conditional inclusion into models using Bayesian kernel machine regression model.

|  | **GroupPIP** | |  |  | **CondPIP** | | | | | |
| --- | --- | --- | --- | --- | --- | --- | --- | --- | --- | --- |
|  | isoflavones | lignans |  | isoflavones | | | |  | lignans | |
|  |  |  |  | DAD | O-DMA | EQU | GEN |  | ENT | END |
| Serum uric acid | **1.00** | **0.01** |  | 0.00 | 0.00 | **1.00** | 0.00 |  | **1.00** | 0.00 |
| Hyperuricemia | **1.00** | **0.73** |  | 0.00 | 0.00 | **1.00** | 0.00 |  | **1.00** | 0.00 |

Abbreviations: DAD, daidzein; O-DMA, O-desmethylangolensin; EQU, equol; GEN, genistein; ETD, enterodiol; ENT, enterolactone; PIP, posterior inclusion probability.

**Table S4** Associations of urinary phytoestrogen biomarkers with the estimated glomerular filtration rate.

|  | β (95% CI) | *P*-value |
| --- | --- | --- |
| Daidzein | 0.17 (-0.14, 0.48) | 0.275 |
| O-Desmethylangolensin | 0.19 (-0.04, 0.42) | 0.112 |
| **Equol** | **0.57 (0.18, 0.97)** | **0.004** |
| Genistein | 0.28 (-0.04, 0.61) | 0.087 |
| Enterodiol | 0.38 (0.06, 0.69) | 0.020 |
| **Enterolactone** | **0.36 (0.06, 0.67)** | **0.019** |

Adjusting for age, sex, race/ethnicity, poverty income ratio, body mass index, educational levels, smoking status, alcohol consumption, recreational physical activity, hypertension, and diabetes.

**Table S5** Associations of urinary phytoestrogen biomarkers with hyperuricemia according to the American College of Rheumatology guidelines in multiple logistic regression models

|  | Crude model | |  | Adjusted model | |
| --- | --- | --- | --- | --- | --- |
|  | OR (95% CI) | *P*-value |  | OR (95% CI) | *P*-value |
| Daidzein |  |  |  |  |  |
| Quartile1 | Ref | Ref |  | Ref | Ref |
| Quartile2 | 1.05 (0.79, 1.40) | 0.727 |  | 1.05 (0.77, 1.43) | 0.750 |
| Quartile3 | 0.91 (0.68, 1.21) | 0.505 |  | 0.84 (0.62, 1.15) | 0.286 |
| Quartile4 | 0.72 (0.53, 0.97) | 0.032 |  | 0.82 (0.59, 1.14) | 0.243 |
| Continuous | 0.90 (0.85, 0.96) | 0.001 |  | 0.92 (0.86, 0.99) | 0.022 |
| O-Desmethylangolensin |  |  |  |  |  |
| Quartile1 | Ref | Ref |  | Ref | Ref |
| Quartile2 | 0.65 (0.49, 0.87) | 0.004 |  | 0.62 (0.46, 0.85) | 0.003 |
| Quartile3 | 0.69 (0.52, 0.92) | 0.012 |  | 0.60 (0.44, 0.82) | 0.001 |
| Quartile4 | 0.57 (0.43, 0.77) | <0.001 |  | 0.62 (0.45, 0.85) | 0.003 |
| Continuous | 0.90 (0.86, 0.94) | <0.001 |  | 0.91 (0.86, 0.96) | 0.001 |
| Equol |  |  |  |  |  |
| Quartile1 | Ref | Ref |  | Ref | Ref |
| Quartile2 | 0.70 (0.53, 0.92) | 0.010 |  | 0.69 (0.51, 0.93) | 0.016 |
| Quartile3 | 0.69 (0.52, 0.91) | 0.008 |  | 0.71 (0.52, 0.96) | 0.025 |
| Quartile4 | 0.33 (0.24, 0.46) | <0.001 |  | 0.35 (0.25, 0.51) | <0.001 |
| Continuous | 0.76 (0.70, 0.83) | <0.001 |  | 0.76 (0.69, 0.84) | <0.001 |
| Genistein |  |  |  |  |  |
| Quartile1 | Ref | Ref |  | Ref | Ref |
| Quartile2 | 0.65 (0.49, 0.88) | 0.006 |  | 0.67 (0.49, 0.93) | 0.015 |
| Quartile3 | 0.96 (0.73, 1.27) | 0.776 |  | 0.99 (0.73, 1.33) | 0.924 |
| Quartile4 | 0.71 (0.53, 0.95) | 0.021 |  | 0.80 (0.58, 1.10) | 0.166 |
| Continuous | 0.94 (0.89, 1.01) | 0.080 |  | 0.98 (0.91, 1.05) | 0.590 |
| Enterodiol |  |  |  |  |  |
| Quartile1 | Ref | Ref |  | Ref | Ref |
| Quartile2 | 0.75 (0.57, 0.99) | 0.041 |  | 0.68 (0.50, 0.92) | 0.013 |
| Quartile3 | 0.74 (0.56, 0.98) | 0.033 |  | 0.72 (0.53, 0.97) | 0.032 |
| Quartile4 | 0.44 (0.32, 0.60) | <0.001 |  | 0.51 (0.36, 0.72) | <0.001 |
| Continuous | 0.88 (0.83, 0.93) | <0.001 |  | 0.90 (0.85, 0.96) | 0.002 |
| Enterolactone |  |  |  |  |  |
| Quartile1 | Ref | Ref |  | Ref | Ref |
| Quartile2 | 0.72 (0.54, 0.94) | 0.018 |  | 0.70 (0.52, 0.94) | 0.018 |
| Quartile3 | 0.58 (0.44, 0.78) | <0.001 |  | 0.54 (0.39, 0.74) | <0.001 |
| Quartile4 | 0.50 (0.37, 0.67) | <0.001 |  | 0.56 (0.40, 0.79) | <0.001 |
| Continuous | 0.86 (0.82, 0.91) | <0.001 |  | 0.87 (0.82, 0.92) | <0.001 |

Adjusting for age, sex, race/ethnicity, poverty income ratio, body mass index, educational levels, smoking status, alcohol consumption, recreational physical activity, hypertension, and diabetes.

**Table S6**. Associations of urinary phytoestrogen biomarkers with serum uric acid concentrations and hyperuricemia risk stratified by sex and survey cycle

|  | Serum uric acid | |  | Hyperuricemia | |
| --- | --- | --- | --- | --- | --- |
|  | β (95% CI) | *P*-value |  | OR (95% CI) | *P*-value |
| Male ^a^ |  |  |  |  |  |
| Daidzein | -0.07 (-0.11, -0.03) | <0.001 |  | 0.93 (0.86, 1.01) | 0.068 |
| O-Desmethylangolensin | -0.06 (-0.09, -0.03) | <0.001 |  | 0.91 (0.85, 0.97) | 0.003 |
| Equol | -0.11 (-0.16, -0.06) | <0.001 |  | 0.82 (0.73, 0.91) | <0.001 |
| Genistein | -0.04 (-0.08, 0.00) | 0.074 |  | 0.99 (0.91, 1.07) | 0.755 |
| Enterodiol | -0.04 (-0.08, 0.00) | 0.067 |  | 0.90 (0.83, 0.97) | 0.006 |
| Enterolactone | -0.06 (-0.10, -0.02) | 0.001 |  | 0.88 (0.82, 0.95) | 0.001 |
| Female ^a^ |  |  |  |  |  |
| Daidzein | -0.05 (-0.08, -0.01) | 0.006 |  | 0.90 (0.82, 0.99) | 0.033 |
| O-Desmethylangolensin | -0.05 (-0.07, -0.02) | <0.001 |  | 0.88 (0.82, 0.95) | 0.001 |
| Equol | -0.10 (-0.14, -0.06) | <0.001 |  | 0.80 (0.70, 0.91) | 0.001 |
| Genistein | -0.03 (-0.06, 0.01) | 0.114 |  | 0.95 (0.87, 1.05) | 0.314 |
| Enterodiol | 0.00 (-0.03, 0.04) | 0.917 |  | 1.01 (0.93, 1.11) | 0.804 |
| Enterolactone | -0.07 (-0.10, -0.03) | 0.000 |  | 0.86 (0.79, 0.93) | <0.001 |
| 2007-2008 ^b^ |  |  |  |  |  |
| Daidzein | -0.08 (-0.11, -0.04) | <0.001 |  | 0.91 (0.83, 0.99) | 0.033 |
| O-Desmethylangolensin | -0.07 (-0.09, -0.04) | <0.001 |  | 0.90 (0.84, 0.96) | 0.001 |
| Equol | -0.12 (-0.17, -0.07) | <0.001 |  | 0.78 (0.69, 0.87) | <0.001 |
| Genistein | -0.06 (-0.09, -0.02) | 0.005 |  | 0.97 (0.89, 1.07) | 0.571 |
| Enterodiol | -0.03 (-0.07, 0.01) | 0.110 |  | 0.93 (0.85, 1.00) | 0.065 |
| Enterolactone | -0.08 (-0.12, -0.04) | <0.001 |  | 0.84 (0.77, 0.9) | <0.001 |
| 2009-2010 ^b^ |  |  |  |  |  |
| Daidzein | -0.04 (-0.08, -0.01) | 0.015 |  | 0.91 (0.84, 0.99) | 0.032 |
| O-Desmethylangolensin | -0.05 (-0.07, -0.02) | 0.001 |  | 0.88 (0.82, 0.94) | <0.001 |
| Equol | -0.09 (-0.13, -0.04) | <0.001 |  | 0.84 (0.75, 0.94) | 0.003 |
| Genistein | -0.01 (-0.05, 0.02) | 0.484 |  | 0.96 (0.88, 1.05) | 0.365 |
| Enterodiol | -0.01 (-0.05, 0.02) | 0.530 |  | 0.95 (0.88, 1.03) | 0.242 |
| Enterolactone | -0.06 (-0.10, -0.03) | <0.001 |  | 0.89 (0.82, 0.96) | 0.002 |

^a^ Adjusting for age, race/ethnicity, poverty income ratio, body mass index, educational levels, smoking status, alcohol consumption, recreational physical activity, hypertension, and diabetes.

^b^ Adjusting for age, sex, race/ethnicity, poverty income ratio, body mass index, educational levels, smoking status, alcohol consumption, recreational physical activity, hypertension, and diabetes.

**Table S7.** Associations of urinary phytoestrogen biomarkers with serum uric acid concentrations and hyperuricemia risk within different subgroups

|  | **Serum uric acid** | |  | **Hyperuricemia** | |
| --- | --- | --- | --- | --- | --- |
|  | **β (95% CI)** | ***P*-value** |  | **OR (95% CI)** | ***P*-value** |
| **Adults with normal weight ^a^** |  |  |  |  |  |
| Daidzein | -0.10 (-0.14, -0.06) | <0.001 |  | 0.78 (0.66, 0.93) | 0.005 |
| O-Desmethylangolensin | -0.07 (-0.10, -0.04) | <0.001 |  | 0.87 (0.77, 0.98) | 0.028 |
| Equol | -0.10 (-0.15, -0.05) | <0.001 |  | 0.78 (0.65, 0.94) | 0.009 |
| Genistein | -0.07 (-0.11, -0.02) | 0.002 |  | 0.84 (0.71, 1.00) | 0.046 |
| Enterodiol | -0.03 (-0.08, 0.01) | 0.174 |  | 0.84 (0.72, 0.98) | 0.023 |
| Enterolactone | -0.06 (-0.10, -0.02) | 0.005 |  | 0.81 (0.71, 0.93) | 0.002 |
| **Adults who were not smoker ^b^** |  |  |  |  |  |
| Daidzein | -0.04 (-0.07, -0.01) | 0.013 |  | 0.93 (0.85, 1.01) | 0.084 |
| O-Desmethylangolensin | -0.06 (-0.08, -0.03) | <0.001 |  | 0.89 (0.83, 0.95) | <0.001 |
| Equol | -0.07 (-0.11, -0.03) | 0.001 |  | 0.86 (0.76, 0.96) | 0.008 |
| Genistein | -0.03 (-0.06, 0.00) | 0.091 |  | 0.97 (0.89, 1.06) | 0.481 |
| Enterodiol | -0.02 (-0.05, 0.02) | 0.385 |  | 0.95 (0.88, 1.03) | 0.245 |
| Enterolactone | -0.07 (-0.11, -0.04) | <0.001 |  | 0.87 (0.80, 0.94) | 0.001 |
| **Adults who were not drinker ^c^** |  |  |  |  |  |
| Daidzein | -0.05 (-0.10, 0.01) | 0.089 |  | 0.94 (0.83, 1.06) | 0.313 |
| O-Desmethylangolensin | -0.05 (-0.09, -0.01) | 0.016 |  | 0.94 (0.85, 1.03) | 0.164 |
| Equol | -0.12 (-0.19, -0.05) | 0.000 |  | 0.87 (0.73, 1.02) | 0.091 |
| Genistein | -0.03 (-0.08, 0.03) | 0.333 |  | 0.96 (0.84, 1.09) | 0.518 |
| Enterodiol | -0.04 (-0.09, 0.02) | 0.172 |  | 0.93 (0.82, 1.04) | 0.192 |
| Enterolactone | -0.08 (-0.13, -0.03) | 0.002 |  | 0.89 (0.79, 0.99) | 0.039 |
| **Adults without history of hypertension ^d^** | | | | | |
| Daidzein | -0.06 (-0.09, -0.03) | <0.001 |  | 0.91 (0.82, 1.01) | 0.063 |
| O-Desmethylangolensin | -0.05 (-0.08, -0.03) | <0.001 |  | 0.89 (0.83, 0.97) | 0.006 |
| Equol | -0.10 (-0.14, -0.06) | <0.001 |  | 0.73 (0.62, 0.85) | <0.001 |
| Genistein | -0.03 (-0.06, 0.01) | 0.109 |  | 1.01 (0.91, 1.12) | 0.813 |
| Enterodiol | -0.02 (-0.06, 0.01) | 0.177 |  | 0.91 (0.82, 1.00) | 0.062 |
| Enterolactone | -0.07 (-0.10, -0.03) | <0.001 |  | 0.87 (0.79, 0.95) | 0.003 |
| **Adults without history of diabetes ^e^** |  |  |  |  |  |
| Daidzein | -0.06 (-0.09, -0.03) | <0.001 |  | 0.91 (0.85, 0.97) | 0.005 |
| O-Desmethylangolensin | -0.06 (-0.08, -0.04) | <0.001 |  | 0.89 (0.85, 0.94) | <0.001 |
| Equol | -0.11 (-0.15, -0.08) | <0.001 |  | 0.78 (0.71, 0.85) | <0.001 |
| Genistein | -0.04 (-0.07, -0.01) | 0.005 |  | 0.97 (0.91, 1.03) | 0.331 |
| Enterodiol | -0.03 (-0.05, 0.00) | 0.052 |  | 0.93 (0.87, 0.99) | 0.026 |
| Enterolactone | -0.07 (-0.10, -0.05) | <0.001 |  | 0.86 (0.81, 0.91) | <0.001 |
| **Adults without history of CKD ^f^** |  |  |  |  |  |
| Daidzein | -0.06 (-0.08, -0.03) | <0.001 |  | 0.92 (0.86, 0.99) | 0.021 |
| O-Desmethylangolensin | -0.06 (-0.08, -0.04) | <0.001 |  | 0.88 (0.84, 0.93) | <0.001 |
| Equol | -0.10 (-0.14, -0.07) | <0.001 |  | 0.81 (0.73, 0.88) | <0.001 |
| Genistein | -0.03 (-0.05, -0.00) | 0.032 |  | 1.01 (0.94, 1.08) | 0.814 |
| Enterodiol | -0.01 (-0.04, 0.02) | 0.433 |  | 0.97 (0.91, 1.03) | 0.358 |
| Enterolactone | -0.07 (-0.10, -0.05) | <0.001 |  | 0.86 (0.81, 0.91) | <0.001 |

^a^ Adjusting for age, sex, race/ethnicity, poverty income ratio, smoking status, alcohol consumption, recreational physical activity, hypertension, and diabetes

^b^ Adjusting for age, sex, race/ethnicity, poverty income ratio, body mass index, alcohol consumption, recreational physical activity, hypertension, and diabetes

^c^ Adjusting for age, sex, race/ethnicity, poverty income ratio, body mass index, smoking status, recreational physical activity, hypertension, and diabetes

^d^ Adjusting for age, sex, race/ethnicity, poverty income ratio, body mass index, smoking status, alcohol consumption, recreational physical activity, and diabetes

^e^ Adjusting for age, sex, race/ethnicity, poverty income ratio, body mass index, smoking status, alcohol consumption, recreational physical activity, and hypertension

^f^ Adjusting for age, sex, race/ethnicity, poverty income ratio, body mass index, smoking status, alcohol consumption, recreational physical activity, hypertension, and diabetes

**Table S8.** Associations of urinary phytoestrogen biomarkers with serum uric acid concentrations and hyperuricemia risk, additionally adjusted for total protein intake

|  | **Serum uric acid** |  |  | **Hyperuricemia** | |
| --- | --- | --- | --- | --- | --- |
|  | **β (95% CI)** | ***P*-value** |  | **OR (95% CI)** | ***P*-value** |
| Daidzein |  |  |  |  |  |
| Quartile1 | Ref | Ref |  | Ref |  |
| Quartile2 | -0.12 (-0.24, 0.01) | 0.065 |  | 0.98 (0.75, 1.29) | 0.900 |
| Quartile3 | -0.20 (-0.33, -0.08) | 0.002 |  | 0.73 (0.55, 0.96) | 0.025 |
| Quartile4 | -0.27 (-0.39, -0.14) | <0.001 |  | 0.75 (0.57, 1.00) | 0.049 |
| Continuous | -0.06 (-0.09, -0.04) | <0.001 |  | 0.91 (0.86, 0.97) | 0.003 |
| O-Desmethylangolensin |  |  |  |  |  |
| Quartile1 | Ref | Ref |  | Ref | Ref |
| Quartile2 | -0.26 (-0.39, -0.14) | <0.001 |  | 0.65 (0.49, 0.85) | 0.002 |
| Quartile3 | -0.36 (-0.49, -0.23) | <0.001 |  | 0.56 (0.43, 0.74) | <0.001 |
| Quartile4 | -0.34 (-0.46, -0.21) | <0.001 |  | 0.56 (0.43, 0.75) | <0.001 |
| Continuous | -0.06 (-0.08, -0.04) | <0.001 |  | 0.89 (0.85, 0.93) | <0.001 |
| Equol |  |  |  |  |  |
| Quartile1 | Ref | Ref |  | Ref | Ref |
| Quartile2 | -0.24 (-0.37, -0.12) | <0.001 |  | 0.69 (0.53, 0.91) | 0.008 |
| Quartile3 | -0.25 (-0.38, -0.12) | <0.001 |  | 0.71 (0.54, 0.93) | 0.014 |
| Quartile4 | -0.48 (-0.61, -0.35) | <0.001 |  | 0.45 (0.33, 0.61) | <0.001 |
| Continuous | -0.11 (-0.14, -0.07) | <0.001 |  | 0.81 (0.74, 0.88) | <0.001 |
| Genistein |  |  |  |  |  |
| Quartile1 | Ref | Ref |  | Ref | Ref |
| Quartile2 | -0.2 (-0.32, -0.07) | 0.002 |  | 0.82 (0.62, 1.08) | 0.162 |
| Quartile3 | -0.10 (-0.23, 0.03) | 0.120 |  | 0.92 (0.7, 1.21) | 0.560 |
| Quartile4 | -0.22 (-0.35, -0.09) | 0.001 |  | 0.80 (0.60, 1.05) | 0.112 |
| Continuous | -0.03 (-0.06, -0.00) | 0.022 |  | 0.97 (0.91, 1.03) | 0.375 |
| Enterodiol |  |  |  |  |  |
| Quartile1 | Ref | Ref |  | Ref | Ref |
| Quartile2 | -0.09 (-0.22, 0.04) | 0.160 |  | 0.76 (0.58, 1.00) | 0.053 |
| Quartile3 | -0.04 (-0.16, 0.09) | 0.579 |  | 0.89 (0.68, 1.17) | 0.401 |
| Quartile4 | -0.09 (-0.22, 0.04) | 0.163 |  | 0.72 (0.54, 0.96) | 0.026 |
| Continuous | -0.02 (-0.05, 0.00) | 0.099 |  | 0.94 (0.89, 0.99) | 0.029 |
| Enterolactone |  |  |  |  |  |
| Quartile1 | Ref | Ref |  | Ref | Ref |
| Quartile2 | -0.21 (-0.34, -0.09) | 0.001 |  | 0.63 (0.48, 0.83) | 0.001 |
| Quartile3 | -0.27 (-0.40, -0.15) | <0.001 |  | 0.58 (0.44, 0.76) | <0.001 |
| Quartile4 | -0.24 (-0.37, -0.11) | <0.001 |  | 0.58 (0.44, 0.78) | <0.001 |
| Continuous | -0.07 (-0.10, -0.05) | <0.001 |  | 0.86 (0.81, 0.91) | <0.001 |

The model was adjusted for age, sex, race/ethnicity, poverty income ratio, body mass index, educational level, smoking status, alcohol consumption, recreational physical activity, hypertension, diabetes, and total protein intake.

**Table S9.** Associations of uncorrected urinary phytoestrogen biomarkers with serum uric acid concentrations and hyperuricemia risk, with urinary creatinine as an adjustment covariate.

|  | | **Serum uric acid** | |  | |  | | **Hyperuricemia** | | | |
| --- | --- | --- | --- | --- | --- | --- | --- | --- | --- | --- | --- |
|  | | **β (95% CI)** | | ***P*-value** | |  | | **OR (95% CI)** | | ***P*-value** | |
| Daidzein | |  | |  | |  | |  | |  | |
| Quartile1 | | Ref | | Ref | |  | | Ref | |  | |
| Quartile2 | | -0.15 (-0.28, -0.03) | | 0.018 | |  | | 0.88 (0.66, 1.16) | | 0.363 | |
| Quartile3 | | -0.23 (-0.36, -0.10) | | 0.001 | |  | | 0.73 (0.55, 0.97) | | 0.032 | |
| Quartile4 | | -0.28 (-0.41, -0.15) | | 0.000 | |  | | 0.73 (0.55, 0.98) | | 0.038 | |
| Continuous | | -0.06 (-0.08, -0.03) | | <0.001 | |  | | 0.92 (0.86, 0.97) | | 0.004 | |
| O-Desmethylangolensin | |  | |  | |  | |  | |  | |
| Quartile1 | | Ref | | Ref | |  | | Ref | | Ref | |
| Quartile2 | | -0.07 (-0.20, 0.06) | | 0.265 | |  | | 0.80 (0.61, 1.06) | | 0.120 | |
| Quartile3 | | -0.28 (-0.41, -0.15) | | 0.000 | |  | | 0.62 (0.46, 0.82) | | 0.001 | |
| Quartile4 | | -0.24 (-0.37, -0.11) | | 0.000 | |  | | 0.65 (0.48, 0.87) | | 0.003 | |
| Continuous | | -0.05 (-0.07, -0.03) | | <0.001 | |  | | 0.90 (0.86, 0.94) | | <0.001 | |
| Equol | |  | |  | |  | |  | |  | |
| Quartile1 | | Ref | | Ref | |  | | Ref | | Ref | |
| Quartile2 | | -0.16 (-0.29, -0.04) | | 0.011 | |  | | 0.76 (0.58, 1.00) | | 0.049 | |
| Quartile3 | | -0.36 (-0.49, -0.23) | | 0.000 | |  | | 0.52 (0.39, 0.69) | | <0.001 | |
| Quartile4 | | -0.47 (-0.61, -0.34) | | 0.000 | |  | | 0.44 (0.33, 0.60) | | <0.001 | |
| Continuous | | -0.10 (-0.13, -0.07) | | <0.001 | |  | | 0.82 (0.75, 0.88) | | <0.001 | |
| Genistein | |  | |  | |  | |  | |  | |
| Quartile1 | | Ref | | Ref | |  | | Ref | | Ref | |
| Quartile2 | | -0.16 (-0.29, -0.03) | | 0.014 | |  | | 0.83 (0.63, 1.11) | | 0.208 | |
| Quartile3 | | -0.10 (-0.23, 0.03) | | 0.114 | |  | | 0.89 (0.67, 1.19) | | 0.436 | |
| Quartile4 | | -0.12 (-0.25, 0.01) | | 0.075 | |  | | 0.96 (0.72, 1.29) | | 0.803 | |
| Continuous | | -0.03 (-0.05, 0.00) | | 0.052 | |  | | 0.98 (0.92, 1.04) | | 0.464 | |
| Enterodiol | |  | |  | |  | |  | |  | |
| Quartile1 | | Ref | | Ref | |  | | Ref | | Ref | |
| Quartile2 | | 0.01 (-0.12, 0.13) | | 0.895 | |  | | 0.89 (0.68, 1.18) | | 0.418 | |
| Quartile3 | | 0.01 (-0.12, 0.14) | | 0.861 | |  | | 0.85 (0.64, 1.12) | | 0.244 | |
| Quartile4 | | 0.00 (-0.13, 0.13) | | 0.998 | |  | | 0.85 (0.63, 1.13) | | 0.257 | |
| Continuous | | -0.02 (-0.04, 0.01) | | 0.256 | |  | | 0.95 (0.89, 1.00) | | 0.051 | |
| Enterolactone | |  | |  | |  | |  | |  | |
| Quartile1 | | Ref | | Ref | |  | | Ref | | Ref | |
| Quartile2 | | -0.11 (-0.24, 0.02) | | 0.085 | |  | | 0.91 (0.7, 1.19) | | 0.503 | |
| Quartile3 | | -0.1 (-0.23, 0.03) | | 0.119 | |  | | 0.77 (0.59, 1.01) | | 0.057 | |
| Quartile4 | | -0.22 (-0.35, -0.09) | | 0.001 | |  | | 0.61 (0.46, 0.82) | | 0.001 | |
| Continuous | | -0.06 (-0.09, -0.04) | | <0.001 | |  | | 0.87 (0.82, 0.92) | | <0.001 | |

The model was adjusted for age, sex, race/ethnicity, poverty income ratio, body mass index, educational level, smoking status, alcohol consumption, recreational physical activity, hypertension, diabetes, and urine creatinine.
